# Supplementary figures and images for: Association between anxiety and depression and all-cause mortality: a 50-year follow-up of the Population Study of Women in Gothenburg, Sweden
Source: BMJ Open. 2023 Nov 21;13(11):e075471. doi: 10.1136/bmjopen-2023-075471 (PMC10668159; doi:10.1136/bmjopen-2023-075471)

## Appendix Figure 1

Flowchart of participants in Population Study of Women in Gothenburg in 1968-69

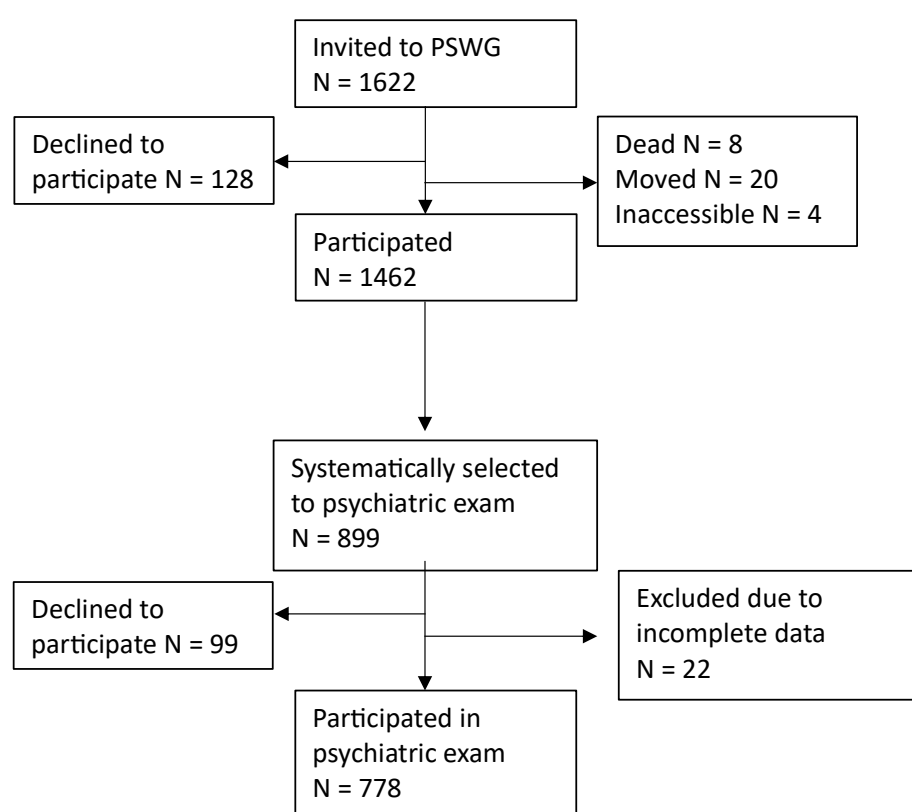

Supplement: Supplementary data [file bmjopen-2023-075471supp001.pdf]
