# Supplementary material for: Association between anxiety and depression and all-cause mortality: a 50-year follow-up of the Population Study of Women in Gothenburg, Sweden
Source: BMJ Open. 2023 Nov 21;13(11):e075471. doi: 10.1136/bmjopen-2023-075471 (PMC10668159; doi:10.1136/bmjopen-2023-075471)
Supplement: Author's manuscript [file bmjopen-2023-075471.draft_revisions.pdf]

BMJ Open is committed to open peer review. As part of this commitment we make the peer review history of every article we publish publicly available.

When an article is published we post the peer reviewers' comments and the authors' responses online. We also post the versions of the paper that were used during peer review. These are the versions that the peer review comments apply to.

The versions of the paper that follow are the versions that were submitted during the peer review process. They are not the versions of record or the final published versions. They should not be cited or distributed as the published version of this manuscript.

BMJ Open is an open access journal and the full, final, typeset and author-corrected version of record of the manuscript is available on our site with no access controls, subscription charges or pay-per-view fees (http://bmjopen.bmj.com).

If you have any questions on BMJ Open's open peer review process please email info.bmjopen@bmj.com

BMJ Open

Association between Anxiety and Depression and all-cause Mortality: a 50-year follow-up of the Population Study of Women in Gothenburg, Sweden

Journal:	*BMJ Open*
Manuscript ID	bmjopen-2023-075471
Article Type:	Original research
Date Submitted by the Author:	09-May-2023

Complete List of Authors:	von Below, Amanda; University of Gothenburg Sahlgrenska Academy, Primary Health Care/School of Public Health and Community Medicine<br>Hällström, Tore; Institute of Neuroscience and Physiology, Sahlgrenska Academy, Department of Psychiatry and Neurochemistry, Gothenburg University<br>Sundh, Valter; University of Gothenburg Sahlgrenska Academy, Primary Health Care/School of Public Health and Community Medicine<br>Björkelund, Cecilia; University of Gothenburg Sahlgrenska Academy, Primary Health Care/School of Public Health and Community Medicine<br>Hange, Dominique; University of Gothenburg Sahlgrenska Academy, Primary Health Care/School of Public Health and Community Medicine

Keywords:	Anxiety disorders < PSYCHIATRY, Depression & mood disorders < PSYCHIATRY, Primary Care < Primary Health Care, Adult psychiatry < PSYCHIATRY, PUBLIC HEALTH

SCHOLARONE™
Manuscripts

I, the Submitting Author has the right to grant and does grant on behalf of all authors of the Work (as defined in the below author licence), an exclusive licence and/or a non-exclusive licence for contributions from authors who are: i) UK Crown employees; ii) where BMJ has agreed a CC-BY licence shall apply, and/or iii) in accordance with the terms applicable for US Federal Government officers or employees acting as part of their official duties; on a worldwide, perpetual, irrevocable, royalty-free basis to BMJ Publishing Group Ltd ("BMJ") its licensees and where the relevant Journal is co-owned by BMJ to the co-owners of the Journal, to publish the Work in this journal and any other BMJ products and to exploit all rights, as set out in our licence.

The Submitting Author accepts and understands that any supply made under these terms is made by BMJ to the Submitting Author unless you are acting as an employee on behalf of your employer or a postgraduate student of an affiliated institution which is paying any applicable article publishing charge ("APC") for Open Access articles. Where the Submitting Author wishes to make the Work available on an Open Access basis (and intends to pay the relevant APC), the terms of reuse of such Open Access shall be governed by a Creative Commons licence – details of these licences and which Creative Commons licence will apply to this Work are set out in our licence referred to above.

Other than as permitted in any relevant BMJ Author's Self Archiving Policies, I confirm this Work has not been accepted for publication elsewhere, is not being considered for publication elsewhere and does not duplicate material already published. I confirm all authors consent to publication of this Work and authorise the granting of this licence.

For peer review only

Association between Anxiety and Depression and all-cause Mortality: a 50-year follow-up of the Population Study of Women in Gothenburg, Sweden

Amanda von Below[1], Tore Hällström[2], Valter Sundh[1], Cecilia Björkelund[1,3], Dominique Hange[1,3]

[1]*Primary Health Care, School of Public Health and Community Medicine, Institute of Medicine, Sahlgrenska Academy, University of Gothenburg, Sweden*

[2]*Neuropsychiatric Epidemiology Unit, Department of Psychiatry and Neurochemistry, Institute of Neuroscience and Physiology, Sahlgrenska Academy, Centre for Aging and Health (AGECAP), University of Gothenburg, Sweden*

[3]*Research, Education, Development & Innovation, Primary Health Care, Region Västra Götaland, Sweden*

Corresponding author
Dominique Hange
Department of Public Health and Community Medicine/Primary Health Care
PO Box 453,
SE-405 30 Gothenburg, Sweden
Phone: +46-31-773 68 49 Fax: +46-31-778 17 04
E-Mail: dominique.hange@allmed.gu.se

Abstract

For peer review only

Objectives: To examine the association between anxiety disorders and/or major depression disorder and all-cause mortality in a 50-year perspective and to examine specific risk and health factors that may mediate such an association.

Design: Observational population study, from 1968-69 until 31st December 2019.

Setting: The Population Study of Women in Gothenburg, Sweden (PSWG) including a representative sample of 1468 women.

Participants: In 1968-69, 899 women from PSWG were systematically selected according to date of birth for a psychiatric investigation, including diagnostic evaluation. Eight hundred (89%) accepted the invitation.

Primary and secondary outcome measures: Associations between all-cause mortality and anxiety disorders (AD), major depressive disorder (MDD) and comorbid AD/MDD with adjustments for potential confounding factors. Differences between the groups concerning health and risk factors and their association with mortality.

Results: When adjusted for age, smoking, physical activity and alcohol consumption, anxiety disorders were associated with all-cause mortality (HR 1.20, 95% CI 1.00-1.44). With further adjustments, the association was non-significant (HR 1.17, 95% CI 0.98-1.41). When examining age during risk time as separate intervals, a significant association between mortality and AD was seen in the age range 65-80 years (HR 1.70, 95% CI 1.26-2.29). In contrast, no associations were found in the younger or older age interval. Among confounding factors, smoking and physical activity were the strongest contributors. The association between smoking and mortality tended to be further increased in the group with anxiety disorders vs. the group without such disorders (HR 2.10, 95% CI 1.60-2.75 and HR 1.82, 95% CI 1.56-2.12, respectively).

Conclusions: This study found an association between anxiety disorders and mortality in women with 50 years of follow-up depending on age, but no association between major depressive disorder and mortality. Further, a higher smoking rate among women with anxiety disorders, and the association between smoking and mortality are undisputed.

Strengths and limitations of this study

For peer review only - http://bmjopen.bmj.com/site/about/guidelines.xhtml

- Strengths include a follow-up time of five decades, a psychiatric investigation based on DSM criteria rather than self-assessed symptoms at baseline, which ensures the accuracy of the disorders, a representative study sample and a high participation rate.
- Limitations are the relatively small sample size and an exclusively female population.

INTRODUCTION

Worldwide, mental disorders are among the top ten leading causes of morbidity. Major depressive disorder (MDD) and anxiety disorders (AD) are the most significant contributors to this, affecting more than half a billion people, and are overrepresented among women.[1,2] The Swedish National Board of Health and Welfare states that persons with mental disorders have an increased risk of incidence as well as mortality in somatic disorders, compared to people without mental illness.[3] However, the association between mental disorders and mortality is still debatable. Denollet et al. followed 5000 healthy Dutch women for ten years.[4] They found that anxiety symptoms predicted premature all-cause and cardiovascular mortality.[4] A later study distinguished between anxiety symptoms and anxiety disorders and followed persons 11-19 years.[5] No prediction of mortality, whether in women or men, was found.[5] A Danish register study with nine years of follow-up showed that anxiety disorders significantly increased mortality risk, both in women and men.[6] Cardiovascular disease and suicide are both potential contributors to increased mortality among people with MDD and AD.[7-9] A variety of factors associated with poor health, such as hypertension, obesity and excess smoking, have also been linked to common mental disorders.[10-14] The challenge remains to determine whether depression/anxiety at baseline is associated with later smoking, or if smoking is associated with later depression/anxiety.[15]

Two meta-analyses from 2015 and 2016 that studied the association between mortality and AD found conflicting results. One of the meta-analyses concluded that there was an association between mortality and AD, while the other found no such association. The diversity in results could be due to several reasons. Including psychiatric inpatients tended to increase the association,[16] while studying a community sample and adjusting for comorbid AD/MDD had the opposite effect.[17] Further, the identification of the disorders differed, with the majority based on medical records in one study[16] and self-reported symptoms in the other.[17] However, the follow-up time was a median of ten years in one of the meta-analyses but less in the other, which limits the evaluation of the long-term effect. MDD, on the other

For peer review only - http://bmjopen.bmj.com/site/about/guidelines.xhtml

hand, has repeatedly been associated with increased all-cause mortality, especially among men.[18-20] In the Stirling County Study, Murphy et al. showed an association between depression and premature death among men (HR 2.6, 95% CI 1.4-4.9) in 10 years of follow-up. However, this was not seen among depressed women (HR 1.4, 95% CI 0.6-3.2).[21]

In 1968, the Prospective Population Study of Women in Gothenburg started, including middle-aged women.[22] Because of its rather extensive content, the repeated follow-up exams and information regarding mortality during more than 50 years, it was possible to study the associations between disorders and mortality from middle age to the elderly. Given the contradictory results in previous research regarding AD and mortality, the aim was primarily to investigate a possible association between all-cause mortality and AD, secondarily MDD and comorbid AD/MDD, respectively. Another aim was to analyse the risk and health factors which mediate such a possible association.

METHODS AND MATERIAL

The Population Study of Women in Gothenburg includes a representative sample of 1468 women aged 38, 46, 50, 54 and 60 years old who were invited to a medical examination in 1968-69. The participation rate was 90.1%.[22] 899 participants were systematically sampled from the four youngest age groups according to birth date and invited for a psychiatric examination. In this subsample, the participation rate was 89% of the invited subjects in the general population. Of those, 22 women were excluded from the present study because of incomplete data due to language difficulties, psychosis or intellectual disability. A comparison between non-participants and participants showed more single women among the non-participants. Still, no differences were found concerning mental disorders or the need for hospitalisation because of them.[23,24] The psychiatric investigation was 1-2 hours long and was conducted using a comprehensive structured questionnaire.

For peer review only - http://bmjopen.bmj.com/site/about/guidelines.xhtml

Anxiety disorders and Major Depressive Disorder at baseline

A psychiatrist performed the psychiatric examination through a face-to-face interview.[25] The findings from the psychiatric investigation were later revised by scrutinising items in the interview questionnaire relevant to a DSM-III MDD diagnosis.[23] Cases of AD was identified from the dataset using algorithms based on the DSM-IV criteria. AD included social phobia, panic disorder, agoraphobia, generalised anxiety disorder (GAD) and obsessive-compulsive disorder (OCD). Identifying cases of posttraumatic stress disorder from items in the dataset was impossible. Specific phobias were excluded due to their generally limited clinical importance.

All-cause mortality rate

Information regarding participants' mortality was collected from the Swedish Cause of Death Register. The mortality rate was observed from the first examination date until the 31st of December 2019.

Statistics

Baseline characteristics were described using chi-square and independent t-test. The odds ratio (OR, confidence interval CI 95%) was used to measure the association between the baseline characteristics in the women with and without AD/MDD. Poisson regression analysis was used to analyse associations with mortality. The hazard ratio (HR, CI 95%) was used to measure the relative risk of death over time in women with AD/MDD compared to women without AD/MDD. The risk time was measured from baseline to the time of death or 31st December 2019. The median follow-up time was 36.9 years (minimum 0.33, maximum 51.3 years). The total risk time was 27260 years and subdivided into quarters of a year in the analyses. When analysing the association between mortality and all AD and all MDD, covariates were sequentially added (Table 1). Still, each covariate was tested in a separate model with age as the only covariate, as small sample sizes limited the number of covariates that could be entered simultaneously (Table 2). An advantage of Poisson models compared to Cox models is that age can be modelled as a continuous time-dependent factor, making the test of interaction with regard to age in risk time much more accessible. Statistical significance was accepted at $p < 0.05$ in all analyses except tests of interaction, which was accepted at $p < 0.10$. SPSS version 29 and Stata version 17 were used to perform statistical analysis.

For peer review only - http://bmjopen.bmj.com/site/about/guidelines.xhtml

For peer review only

Potential confounders

To reduce the effect of potential confounders, the following covariates were used: age (in years), education (over or under six years), economic status (defined as annual income divided by number of family members), alcohol consumption (five level item, from never to everyday use of wine, beer and liquor, separately), smoking (current or non-current), physical activity (four level item, from a sedentary leisure time to regular physical exercise)[26] and BMI (weight in kilogram divided with the square of height in meters). All covariates except age were dichotomised in the regression models. These data were collected in interviews or measured.

Ethical considerations

The Regional Ethical Committee in Gothenburg approved the study (258-16 T853-16). The research was conducted following the World Medical Association Declaration of Helsinki, and all participants provided written informed consent.

RESULTS

AD, MDD and comorbid AD/MDD

A total of 778 participants were included in the study. One hundred sixty participants (20.6%) had one or more AD, and 57 (7.3%) had MDD. Of these, 25 participants (3.2%) had comorbid AD/MDD. The numbers of those with AD solely and MDD solely are presented in Table 1.

The distribution of disorders among AD was GAD n=71 (9.1%), agoraphobia n=67 (8.6%), panic disorder n=58 (7.4%), social phobia n=51 (6.6%) and obsessive-compulsive disorder (OCD) n=28 (3.6%). Of the 135 participants with AD, 85 had one disorder, 48 had two, 17 had three, 7 had four, and 3 had all five disorders.

Baseline characteristics

Baseline characteristics are shown in Table 1. Participants with AD solely (i.e. excluding participants with comorbid AD/MDD) did, to a greater extent, smoke and consume more alcohol (beer) than those without any disorder. Participants with comorbid AD/MDD had significantly more sedentary leisure time and lower economic status. Participants with MDD were, to a lesser extent, married.

For peer review only - http://bmjopen.bmj.com/site/about/guidelines.xhtml

For peer review only

Table 1. Baseline characteristics of the different groups of women with AD solely, MDD solely, comorbid AD/MDD and no AD or MDD, at the examination year 1968-69.

**Characteristics, Mean (SD)**	AD solely n=135	MDD solely n=32	Comorbid AD/MDD n=25	No AD or MDD n=586
Age (years)	47.6 (4.4)	47.8 (5.1)	47.2 (5.3)	47.9 (4.5)
Economic status[1], 1000 SEK	16.2 (8.5)	16.6 (12.2)	**12.0 (4.4)** *	16.1 (9.8)
Number of cigarettes per day among smokers	10.5 (6.0)	**14.1 (7.9)** *	11.2 (8.3)	10.2 (6.0)
BMI kg/m2	24.4 (4.3)	23.7 (2.6)	24.2 (3.4)	24.1 (3.7)
**Characteristics, Prevalence (%)**				
Education six years or more, n (%)	32 (23.7)	6 (18.8)	3 (12.0)	153 (26.1)
Marital status, married	102 (77.9)	**19 (59.4)** *	22 (88)	483 (82.4)
Physical activity Sedentary leisure time, n (%)	27 (20.6)	4 (12.5)	**8 (32.0)** *	95 (16.2)
Smoking, n (%)	**71 (52.6)** **	16 (50.0)	13 (52.0)	226 (38.8)
Economic status (in 10% lowest income category) n (%)	13 (9.7)	2 (6.3)	1 (4.2)	55 (9.5)
Regular alcohol use, n (%)				
Beer	**49 (37.4)** **	5 (15.6)	5 (20)	162 (27.6)
Wine	8 (6.1)	0 (0)	0 (0)	33 (5.6)
Spirits	1 (0.8)	0 (0)	0 (0)	8 (1.4)
BMI >30	13 (9.9)	1 (3.1)	1 (4.0)	44 (7.5.)
BMI <19	8 (6.1)	1 (3.1)	2 (8.0)	28 (4.8)

* p <0.05 ** p <0.01
[1]Annual income divided by number of family members
The significance is tested for the chosen group compared to the group with neither AD nor MDD.

Smoking, beer consumption and physical activity

Having AD significantly increased the odds of being a smoker (OR 1.78, CI 1.21-2.60, p<0.01) and regularly consuming beer (OR 1.58, CI 1.06-2.35, p<0.05), compared to not having any of the disorders. Having MDD increased the odds of being a heavy smoker (>13 cigarettes/day) (OR 2.85, CI 1.22-6.65, p<0.05). Having comorbid AD/MDD significantly increased the odds of having a sedentary leisure time (OR 2.47, CI 1.03-5.89, p<0.05).

All cases of AD and MDD and all-cause mortality rates

Of the total 778 participants, 720 died before the 1st of January 2020. A multivariate regression survival model was used to examine the associations between all cases of AD, all

For peer review only - http://bmjopen.bmj.com/site/about/guidelines.xhtml

cases of MDD and mortality (Table 2). A significant association was found between AD and mortality when adjusted for age. Adding smoking, physical activity and alcohol consumption to the analysis weakened the association somewhat, but it was still significant. In the full model, this association did not reach significance level. In a corresponding analysis of all cases of MDD, there was a significant association when adjusted for age.

Table 2. Associations between all cases of AD, all cases of MDD and mortality rate in different adjustment models.

	**AD n=160**	**MDD n=57**
**Covariates**	HR (95% CI)	
Age	**1.25 (1.04-1.49) ***	**1.21 (1.11-1.12) ***
Age, smoking, physical activity, and alcohol use (beer)	**1.20 (1.00-1.44) ***	1.18 (0.90-1.56)
All of the above, economic status, married, education, BMI>30 and BMI<19	1.17 (0.98-1.41)	1.19 (0.90-1.57)
All of the above and depression	1.16 (0.96-1.39)	-
All of the above and anxiety	-	1.15 (0.86-1.52)

Bold figures, statistically significant

When restricting the analysis (full model including all AD and all MDD) to deaths occurring before the age of 80, AD became a significant risk factor (HR 1.49, CI 1.13-1.98, $p<0.01$). This association was even stronger in the age range of 65-80 years (HR 1.70, CI 1.26-2.29, $p<0.001$). To test the significance of interaction for the effect of AD on mortality in different age intervals, one model with three separate AD-effect parameters was calculated for the age groups 38-64, 65-79 and 80-101. This model was compared to a base model assuming constant AD-effect over the ages. The difference in overall model Chi-square between the two Poisson regression models was 8.2 with df=2 and p=0.02.

An age-adjusted model with only two AD-effect parameters, the first for age 65-79 and the second for the younger and older age intervals pooled, yielded Chi-square= 9.3, df=1 and p=0.002 compared to the base model. A fully adjusted model yielded Chi-square=7.94, df=1 and p=0.005.

No associations were seen when analysing MDD and all-cause mortality.

AD solely, MDD solely, comorbid AD/MDD and all-cause mortality rates

For peer review only

As seen in Table 3, AD solely was significantly associated with mortality when adjusted for age and smoking. A corresponding trend was seen in comorbid AD/MDD.

Table 3. Associations between covariates and the mortality rate in the total sample and the groups with AD solely, MDD solely, and comorbid AD/ADD. All models include age.

	Total sample n=778	AD solely n=135	MDD solely n=32	Comorbid AD/MDD n=25
Covariate	HR (95% CI)	HR (95% CI)	HR (95% CI)	HR (95% CI)
1. Age	**1.13 (1.12-1.14) *** **	**1.13 (1.11-1.15) *** **	**1.14 (1.09-1.19) *** **	**1.17 (1.11-1.23) *** **
2. Education ≥ 6 years	0.88 (0.74-1.05)	1.00 (0.62-1.60)	1.20 (0.46-3.19)	0.30 (0.04-2.13)
3. Physical activity: Regular exercise	**0.73 (0.63-0.85) *** **	0.71 (0.49-1.04)	0.79 (0.38-1.66)	1.02 (0.40-2.63)
4. Smoking	**1.82 (1.56-2.13) *** **	**1.86 (1.24-2.79) ** **	2.12 (0.95-4.72)	**5.18 (1.57-17.14) ** **
5. Beer consumption	0.93 (0.84-1.03)	0.83 (0.66-1.05)	0.70 (0.38-1.29)	1.85 (0.76-4.49)
6. BMI<19	**1.66 (1.20-2.31) *** **	2.13 (0.98-4.62)	1.57 (0.17-14.07)	2.38 (0.19-29.8)
7. BMI>30	1.31 (1.00-1.72)	0.88 (0.47-1.67)	2.07 (0.25-16.88)	0.60 (0.07-4.86)
8. Married	1.00 (0.84-1.22)	0.97 (0.62-1.50)	1.29 (0.54-3.07)	1.08 (0.30-3.97)
9. Economic status (lowest decentile)	**1.37 (1.07-1.77) * **	1.43(0.82-2.49)	0.69 (0.22-2.23)	0.37 (0.05-2.86)

Tests of interaction

In a regression analysis, when adjusted for age, the increased mortality risk associated with smoking did increase further among those having AD, compared to not having AD, HR 2.28 and 1.71, but with overlapping 95% CIs, 1.64-3.17 and 1.44-2.02, respectively, p= 0.096. The corresponding difference in mortality risk associated with regular physical activity was negligible, with HR 0.77 (95% CI = 0.57-1.03) and HR 0.73 (95% CI = 0.62-0.86), p= 0.95.

For peer review only - http://bmjopen.bmj.com/site/about/guidelines.xhtml

DISCUSSION

Main findings

In this representative sample of middle-aged women studied at baseline and followed up over 50 years, we found that at baseline, women with AD solely were more often smokers and more often consumed beer compared to those without AD or MDD. Women with MDD solely were to a lesser extent married than women without AD or MDD. Women with comorbid AD/MDD had more often a poor family economy and a sedentary lifestyle.
Women with AD (all cases) had a significantly increased mortality rate when the covariates age, smoking, physical activity and beer consumption were included in the model. This association did not reach significance level in the full model. Women with MDD (all cases) showed a non-significant trend toward increased mortality rate in both models.
When only deaths in the ages 65-80 years were included, we found a strong association between AD (all cases) and increased mortality rate (full model, $p<0.001$). In comparison, no associations at all were found for both the age intervals 38-64 and 80-101, resulting in a significant interaction effect for AD-Age-Mortality. No similar pattern was observed concerning the interaction between MDD-Age-Mortality.

Comparison with other studies

The lack of significant overall association between MDD and all-cause mortality is in contrast to previous findings,[18-20] with some exceptions. Holwerda et al. did find an association, but only among elderly men.[20] Eaton et al., on the other hand, did not find any association, and the lack of this is suggested to be primarily due to a long follow-up time (27 years).
Symptoms of MDD could be disguised signs of poor health. In the short term, this could lead to an increase in mortality risk that later would subside.[27] However, if this were applicable in our study, it would have been revealed in our sample when analysing time intervals, which was not the case.
Our sample of women with MDD may have been too small, which may explain the lack of significant findings in this group. It is notable, however, that in most of our analyses of MDD and mortality, the hazard ratios were similar to those in the analyses of AD. For example, in the 80-101 age range the hazard ratio for MMD was 1.39 (95% CI 0.98-1.98).

The finding of a reduced association between AD and mortality rate when the full model was applied is consistent with previous research.[17,19,27] The increased mortality rate among women

For peer review only - http://bmjopen.bmj.com/site/about/guidelines.xhtml

with AD in the age range 65-80 is not readily comparable to earlier studies due to the difference in follow-up time. One interpretation of this finding could be that AD is associated with mortality only in ages >65 years. This contradicts previous results, where AD in older women has not been associated with increased mortality but concurs with our finding of no association after age 80. [20,28] Another interpretation is that AD that lasts for an extended period of time, increases vulnerability and may impede the ability of individuals to manage their health and access necessary medical care, which can further contribute to an increased morbidity and mortality risk.

Smoking is an established risk factor for mortality. The association between smoking and mortality tended to increase further in the presence of AD and comorbid AD/MDD. A different pattern in smoking habits could explain this. We found that smoking was more common among women with AD. Another study reported that persons with AD, to a lesser extent, cease daily smoking. If they do, the duration of smoking is longer.[29] Our study did not investigate the exposure time. Still, if our sample is comparable to that study, and the women had a longer duration of smoking, this could be one possible contributing cause to the late increase in mortality.

A meta-analysis of smoking and mortality found that the mortality rate decreased among female smokers after 75 or 80 years.[30] This is consistent with our findings of reduced mortality after 80 years among women with AD, where 53% were smoking at baseline. One possible explanation is that after the age of 80 years, subjects susceptible to harmful smoking effects may have died already. Another possible reason might be that after the age of 80 years, other causes of death increase and compete with those related to smoking and AD.

Strengths and weaknesses

The main strengths of this study are the longitudinal design and the almost unparalleled length of follow-up time amounting to five decades. Other strengths are the representative and well-studied sample of middle-aged women in Gothenburg and the high participation rate.

Also, many studies investigating AD and MDD have used various self-administrated questionnaires, measuring symptomatology rather than the frequency of a disorder. This could potentially over- or underdiagnose the psychopathology, and the reported symptoms could

For peer review only - http://bmjopen.bmj.com/site/about/guidelines.xhtml

originate from a somatic condition. In this study, however, the disorder was assessed by a psychiatrist, making the results more accurate. Furthermore, studies tend to investigate associations either among persons with AD or MDD, which could be misleading due to the high comorbidity. In contrast, in the current study the participants with comorbid AD/MDD were examined as a separate group.

The study has some limitations. Firstly, the research sample was relatively small, especially regarding the group having MDD and comorbid AD/MDD. Secondly, the study sample included only women. Lastly, we studied the point prevalence of AD and MDD and not the lifetime prevalence. However, the onset of AD is, above all, in early adolescence to young adulthood and often has a long duration, which is why we can consider that the participants who meet the criterion for AD in middle age have had the disorder for a long time. Correspondingly, most of those with the preponderance to incur the condition should have received it by the time of the baseline examination. The same is true for MDD, which has a median onset in young adulthood. [31]

The significance of the study, unanswered questions and future research

This study adds to the previous findings showing that AD is not convincingly associated with mortality over the entire age interval studied. The same applies to comorbid AD/MDD. The MDD group was small, however, and the results concerning MDD and mortality are therefore inconclusive. The increase in mortality among those with AD aged 65-80 years is a novel finding that needs to be confirmed in future studies. Considering the high prevalence of comorbid AD/MDD cases, future studies on the association between mortality and AD or MDD should include both disorders. Future research should also focus on effective methods to promote a healthier lifestyle among persons with mental disorders especially regarding cessation of smoking and reduction of heavy alcohol consumption to counteract the increased likelihood of developing comorbid medical conditions such as cardiovascular disease.

Funding

This work was supported by Grants from the Swedish Research Council for Health, Working Life and Welfare—FORTE 2007-1958 and grants from the Swedish State under the agreement between the Swedish Government and the county council, the ALF agreement GBG-68771. This research received no specific grant from any public, commercial or not-for-profit sector funding agency.

For peer review only - http://bmjopen.bmj.com/site/about/guidelines.xhtml

Competing interests

None declared.

Each author's contribution

The study was conducted as part of AvBs intern research project. DH, TH and CB supervised the work. AvB analysed the data and prepared the draft manuscript with the support of all the other authors, DH, TH and CB. VS devised the statistical analysis and contributed to the interpretation of it. All the authors approved the final manuscript and revision.

Data sharing statement

Data are available on reasonable request. All data relevant to the study are included in the article.

REFERENCES

1. GBD 2019 Mental Disorders Collaborators. Global, regional, and national burden of 12 mental disorders in 204 countries and territories, 1990-2019: a systematic analysis for the Global Burden of Disease Study 2019. *Lancet Psychiatry*. 2022;9:137-150. doi:10.1016/S2215-0366(21)00395-3
2. Dattani S, Ritchie H. Roser M. "Mental Health". Published online at OurWorldInData.org. 2021.Retrieved from: 'https://ourworldindata.org/mental-health' [Online Resource] May 9, 2023
3. National Board of Health and Welfare, Sweden. Evaluation of care at depression and anxiety disorders. National guidelines – Evaluation Main report with areas for improvement (Socialstyrelsen. Utvärdering av vård vid depression och ångestsyndrom Nationella riktlinjer – Utvärdering. Huvudrapport med förbättringsområden) https://www.socialstyrelsen.se/globalassets/sharepointdokument/artikelkatalog/nationella-riktlinjer/2019-5-12.pdf. 2019.
4. Denollet J, Maas K, Knottnerus A, Keyzer JJ, Pop VJ. Anxiety predicted premature all-cause and cardiovascular death in a 10-year follow-up of middle-aged women. *Journal of clinical epidemiology*. 2009;62:452-456. doi:10.1016/j.jclinepi.2008.08.006
5. Saeed Mirza S, Ikram MA, Hofman A, Tiemeier H. Anxiety does not predict mortality. A population-based study. *World Psychiatry*. 2015;14:103-104. doi:10.1002/wps.20192
6. Meier SM, Mattheisen M, Mors O, Mortensen PB, Laursen TM, Penninx BW. Increased mortality among people with anxiety disorders: total population study. *Br J Psychiatry*. 2016;209:216-221. doi:10.1192/bjp.bp.115.171975

For peer review only - http://bmjopen.bmj.com/site/about/guidelines.xhtml

7. Roest AM, Martens EJ, de Jonge P, Denollet J. Anxiety and risk of incident coronary heart disease: a meta-analysis. *J Am Coll Cardiol*. 2010;56:38-46. doi:10.1016/j.jacc.2010.03.034
8. Van der Kooy K, van Hout H, Marwijk H, Marten H, Stehouwer C, Beekman A. Depression and the risk for cardiovascular diseases: systematic review and meta analysis. *Int J Geriatr Psychiatry*. 2007;22:613-626. doi:10.1002/gps.1723
9. Moitra M, Santomauro D, Degenhardt L, et al. Estimating the risk of suicide associated with mental disorders: A systematic review and meta-regression analysis. *J Psychiatr Res*. 2021;137:242-249. doi:10.1016/j.jpsychires.2021.02.053
10. Luppino FS, de Wit LM, Bouvy PF, et al. Overweight, obesity, and depression: a systematic review and meta-analysis of longitudinal studies. *Arch Gen Psychiat*. 2010;67:220-9. doi:10.1001/archgenpsychiatry.2010.2
11. Konttinen H, van Strien T, Mannisto S, Jousilahti P, Haukkala A. Depression, emotional eating and long-term weight changes: a population-based prospective study. *Int J Behav Nutr Phys Act*. 2019;16:28. doi:10.1186/s12966-019-0791-8
12. Brumpton B, Langhammer A, Romundstad P, Chen Y, Mai XM. The associations of anxiety and depression symptoms with weight change and incident obesity: The HUNT Study. *Int J Obes (Lond)*. 2013;37:1268-74. doi:10.1038/ijo.2012.204
13. Lim LF, Solmi M, Cortese S. Association between anxiety and hypertension in adults: A systematic review and meta-analysis. *Neurosci Biobehav Rev*. 2021;131:96-119. doi:10.1016/j.neubiorev.2021.08.031
14. Weinberger AH, Chaiton MO, Zhu J, Wall MM, Hasin DS, Goodwin RD. Trends in the Prevalence of Current, Daily, and Nondaily Cigarette Smoking and Quit Ratios by Depression Status in the U.S.: 2005-2017. *Am J Prev Med*. 2020;58:691-698. doi:10.1016/j.amepre.2019.12.023
15. Fluharty M, Taylor AE, Grabski M, Munafo MR. The Association of Cigarette Smoking With Depression and Anxiety: A Systematic Review. *Nicotine Tob Res*. 2017;19:3-13. doi:10.1093/ntr/ntw140
16. Walker ER, McGee RE, Druss BG. Mortality in mental disorders and global disease burden implications: a systematic review and meta-analysis. *JAMA Psychiatry*. 2015;72:334-41. doi:10.1001/jamapsychiatry.2014.2502
17. Miloyan B, Bulley A, Bandeen-Roche K, Eaton WW, Goncalves-Bradley DC. Anxiety disorders and all-cause mortality: systematic review and meta-analysis. *Soc Psychiatry Psychiatr Epidemiol*. 2016;51:1467-1475. doi:10.1007/s00127-016-1284-6
18. van Dijk MR, Utens EM, Dulfer K, et al. Depression and anxiety symptoms as predictors of mortality in PCI patients at 10 years of follow-up. *Eur J Prev Cardiol*. 2016;23:552-558. doi:10.1177/2047487315571889
19. Mykletun A, Bjerkeset O, Overland S, Prince M, Dewey M, Stewart R. Levels of anxiety and depression as predictors of mortality: the HUNT study. *Br J Psychiatry*. 2009;195:118-125. doi:10.1192/bjp.bp.108.054866
20. Holwerda TJ, Schoevers RA, Dekker J, Deeg DJ, Jonker C, Beekman AT. The relationship between generalized anxiety disorder, depression and mortality in old age. *Int J Geriatr Psychiatry*. 2007;22:241-249. doi:10.1002/gps.1669
21. Murphy JM, Gilman SE, Lesage A, et al. Time trends in mortality associated with depression: findings from the Stirling County study. *Can J Psychiatry*. 2010;55:776-783. doi:10.1177/070674371005501205

For peer review only

22. Bengtsson C, Blohme G, Hallberg L, et al. The study of women in Gothenburg 1968-1969-a population study. General design, purpose and sampling results. *Acta medica Scandinavica*. 1973;193:311-318.
23. Hällström T. Point prevalence of major depressive disorder in a Swedish urban female population. *Acta Psychiatr Scand*. 1984;69:52-9. doi:10.1111/j.1600-0447.1984.tb04516.x
24. Hällström T. *Mental disorder and sexuality in the climacteric : a study in psychiatric epidemiology*. Diss. Göteborg : Univ.; 1973.
25. Åsberg M, Montgomery SA, Perris C, Schalling D, Sedvall G. A comprehensive psychopathological rating scale. *Acta Psychiatr Scand Suppl*. 1978:5-27. doi:10.1111/j.1600-0447.1978.tb02357.x
26. Saltin B, Grimby G. Physiological analysis of middle-aged and old former athletes. Comparison with still active athletes of the same ages. *Circulation*. 1968;38:1104-1115. doi:10.1161/01.cir.38.6.1104
27. Eaton WW, Roth KB, Bruce M, et al. The relationship of mental and behavioral disorders to all-cause mortality in a 27-year follow-up of 4 epidemiologic catchment area samples. *American journal of epidemiology*. 2013;178:1366-77. doi:10.1093/aje/kwt219
28. van Hout HP, Beekman AT, de Beurs E, et al. Anxiety and the risk of death in older men and women. *Br J Psychiatry*. 2004;185:399-404. doi:10.1192/bjp.185.5.399
29. Lawrence D, Considine J, Mitrou F, Zubrick SR. Anxiety disorders and cigarette smoking: Results from the Australian Survey of Mental Health and Wellbeing. *Aust N Z J Psychiatry*. 2010;44:520-527. doi:10.3109/00048670903571580
30. Gellert C, Schottker B, Brenner H. Smoking and all-cause mortality in older people: systematic review and meta-analysis. *Arch Intern Med*. 2012;172:837-844. doi:10.1001/archinternmed.2012.1397
31. Solmi M, Radua J, Olivola M, et al. Age at onset of mental disorders worldwide: large-scale meta-analysis of 192 epidemiological studies. *Mol Psychiatry*. 2022;27:281-295. doi:10.1038/s41380-021-01161-7

For peer review only

Reporting checklist for cohort study.

Based on the STROBE cohort guidelines.

Instructions to authors

Complete this checklist by entering the page numbers from your manuscript where readers will find each of the items listed below.

Your article may not currently address all the items on the checklist. Please modify your text to include the missing information. If you are certain that an item does not apply, please write "n/a" and provide a short explanation.

Upload your completed checklist as an extra file when you submit to a journal.

In your methods section, say that you used the STROBE cohortreporting guidelines, and cite them as:

von Elm E, Altman DG, Egger M, Pocock SJ, Gotzsche PC, Vandenbroucke JP. The Strengthening the Reporting of Observational Studies in Epidemiology (STROBE) Statement: guidelines for reporting observational studies.

		Reporting Item	Page Number
**Title and abstract**			
Title	#1a	Indicate the study's design with a commonly used term in the title or the abstract	1
Abstract	#1b	Provide in the abstract an informative and balanced summary of what was done and what was found	2
**Introduction**			
Background / rationale	#2	Explain the scientific background and rationale for the investigation being reported	3
Objectives	#3	State specific objectives, including any prespecified hypotheses	4
**Methods**			
Study design	#4	Present key elements of study design early in the paper	4

For peer review only

Setting	#5	Describe the setting, locations, and relevant dates, including periods of recruitment, exposure, follow-up, and data collection	4
Eligibility criteria	#6a	Give the eligibility criteria, and the sources and methods of selection of participants. Describe methods of follow-up.	4
Eligibility criteria	#6b	For matched studies, give matching criteria and number of exposed and unexposed	n/a
Variables	#7	Clearly define all outcomes, exposures, predictors, potential confounders, and effect modifiers. Give diagnostic criteria, if applicable	5
Data sources / measurement	#8	For each variable of interest give sources of data and details of methods of assessment (measurement). Describe comparability of assessment methods if there is more than one group. Give information separately for for exposed and unexposed groups if applicable.	5
Bias	#9	Describe any efforts to address potential sources of bias	n/a
Study size	#10	Explain how the study size was arrived at	4
Quantitative variables	#11	Explain how quantitative variables were handled in the analyses. If applicable, describe which groupings were chosen, and why	5
Statistical methods	#12a	Describe all statistical methods, including those used to control for confounding	
5			
Statistical methods	#12b	Describe any methods used to examine subgroups and interactions	5
Statistical methods	#12c	Explain how missing data were addressed	5
Statistical methods	#12d	If applicable, explain how loss to follow-up was addressed	5
Statistical methods	#12e	Describe any sensitivity analyses	
5			

Results

For peer review only

For peer review only - http://bmjopen.bmj.com/site/about/guidelines.xhtml

Participants	#13a	Report numbers of individuals at each stage of study—eg numbers potentially eligible, examined for eligibility, confirmed eligible, included in the study, completing follow-up, and analysed. Give information separately for for exposed and unexposed groups if applicable.	6
Participants	#13b	Give reasons for non-participation at each stage	5
Participants	#13c	Consider use of a flow diagram	
n/a			
Descriptive data	#14a	Give characteristics of study participants (eg demographic, clinical, social) and information on exposures and potential confounders. Give information separately for exposed and unexposed groups if applicable.	6
Descriptive data	#14b	Indicate number of participants with missing data for each variable of interest	
n/a			
Descriptive data	#14c	Summarise follow-up time (eg, average and total amount)	
6			
Outcome data	#15	Report numbers of outcome events or summary measures over time. Give information separately for exposed and unexposed groups if applicable.	
7			
Main results	#16a	Give unadjusted estimates and, if applicable, confounder-adjusted estimates and their precision (eg, 95% confidence interval). Make clear which confounders were adjusted for and why they were included	8
Main results	#16b	Report category boundaries when continuous variables were categorized	n/a
Main results	#16c	If relevant, consider translating estimates of relative risk into absolute risk for a meaningful time period	
8			

For peer review only

Other analyses	#17	Report other analyses done—eg analyses of subgroups and interactions, and sensitivity analyses	n/a
**Discussion**			
Key results	#18	Summarise key results with reference to study objectives	10
Limitations	#19	Discuss limitations of the study, taking into account sources of potential bias or imprecision. Discuss both direction and magnitude of any potential bias.	12
Interpretation	#20	Give a cautious overall interpretation considering objectives, limitations, multiplicity of analyses, results from similar studies, and other relevant evidence.	12
Generalisability	#21	Discuss the generalisability (external validity) of the study results	12
**Other Information**			
Funding	#22	Give the source of funding and the role of the funders for the present study and, if applicable, for the original study on which the present article is based	12

The STROBE checklist is distributed under the terms of the Creative Commons Attribution License CC-BY. This checklist was completed on 09. May 2023 using https://www.goodreports.org/, a tool made by the EQUATOR Network in collaboration with Penelope.ai

For peer review only

BMJ Open

Association between Anxiety and Depression and all-cause Mortality: a 50-year follow-up of the Population Study of Women in Gothenburg, Sweden

Journal:	*BMJ Open*
Manuscript ID	bmjopen-2023-075471.R1
Article Type:	Original research
Date Submitted by the Author:	03-Oct-2023
Complete List of Authors:	von Below, Amanda; University of Gothenburg Sahlgrenska Academy, Primary Health Care/School of Public Health and Community Medicine<br>Hällström, Tore; Institute of Neuroscience and Physiology, Sahlgrenska Academy, Department of Psychiatry and Neurochemistry, Gothenburg University<br>Sundh, Valter; University of Gothenburg Sahlgrenska Academy, Primary Health Care/School of Public Health and Community Medicine<br>Björkelund, Cecilia; University of Gothenburg Sahlgrenska Academy, Primary Health Care/School of Public Health and Community Medicine<br>Hange, Dominique; University of Gothenburg Sahlgrenska Academy, Primary Health Care/School of Public Health and Community Medicine
**Primary Subject Heading**:	General practice / Family practice
Secondary Subject Heading:	Mental health
Keywords:	Anxiety disorders < PSYCHIATRY, Depression & mood disorders < PSYCHIATRY, Primary Care < Primary Health Care, Adult psychiatry < PSYCHIATRY, PUBLIC HEALTH

SCHOLARONE™ Manuscripts

I, the Submitting Author has the right to grant and does grant on behalf of all authors of the Work (as defined in the below author licence), an exclusive licence and/or a non-exclusive licence for contributions from authors who are: i) UK Crown employees; ii) where BMJ has agreed a CC-BY licence shall apply, and/or iii) in accordance with the terms applicable for US Federal Government officers or employees acting as part of their official duties; on a worldwide, perpetual, irrevocable, royalty-free basis to BMJ Publishing Group Ltd ("BMJ") its licensees and where the relevant Journal is co-owned by BMJ to the co-owners of the Journal, to publish the Work in this journal and any other BMJ products and to exploit all rights, as set out in our licence.

The Submitting Author accepts and understands that any supply made under these terms is made by BMJ to the Submitting Author unless you are acting as an employee on behalf of your employer or a postgraduate student of an affiliated institution which is paying any applicable article publishing charge ("APC") for Open Access articles. Where the Submitting Author wishes to make the Work available on an Open Access basis (and intends to pay the relevant APC), the terms of reuse of such Open Access shall be governed by a Creative Commons licence – details of these licences and which Creative Commons licence will apply to this Work are set out in our licence referred to above.

Other than as permitted in any relevant BMJ Author's Self Archiving Policies, I confirm this Work has not been accepted for publication elsewhere, is not being considered for publication elsewhere and does not duplicate material already published. I confirm all authors consent to publication of this Work and authorise the granting of this licence.

For peer review only

Association between Anxiety and Depression and all-cause Mortality: a 50-year follow-up of the Population Study of Women in Gothenburg, Sweden

Amanda von Below[1], Tore Hällström[2], Valter Sundh[1], Cecilia Björkelund[1,3],Dominique Hange[1,3]

[1]*Primary Health Care, School of Public Health and Community Medicine, Institute of Medicine, Sahlgrenska Academy, University of Gothenburg, Sweden*

[2]*Neuropsychiatric Epidemiology Unit, Department of Psychiatry and Neurochemistry, Institute of Neuroscience and Physiology, Sahlgrenska Academy, Centre for Aging and Health (AGECAP), University of Gothenburg, Sweden*

[3]*Research, Education, Development & Innovation, Primary Health Care, Region Västra Götaland, Sweden*

Corresponding author

Dominique Hange

Department of Public Health and Community Medicine/Primary Health Care

PO Box 453,

SE-405 30 Gothenburg, Sweden

Phone: +46-31-773 68 49 Fax: +46-31-778 17 04

E-Mail: dominique.hange@allmed.gu.se

For peer review only

For peer review only - http://bmjopen.bmj.com/site/about/guidelines.xhtml

Abstract

Objectives: To examine the association between anxiety disorders and/or major depression disorder and all-cause mortality in a 50-year perspective and to examine specific risk and health factors that may influence such an association.

Design: Observational population study, from 1968-69 until 31st December 2019.

Setting: The Population Study of Women in Gothenburg, Sweden (PSWG).

Participants: In 1968-69, 899 (out of in total 1462) women from PSWG were systematically selected according to date of birth for a psychiatric investigation, including diagnostic evaluation. Eight hundred (89%) accepted the invitation. Twenty-two women were excluded. Of the 778 included, 135 participants (17.4 %) had solely anxiety disorders (AD), 32 (4.1%) had solely major depressive disorder (MDD) and 25 (3.2%) had comorbid AD/MDD.

Primary and secondary outcome measures: Associations between anxiety disorders (AD), major depressive disorder (MDD) and comorbid AD/MDD and all-cause mortality with adjustments for potential confounding factors. Differences between the groups concerning health and risk factors and their association with mortality.

Results: In a fully-adjusted model, anxiety disorders were non-significantly associated with all-cause mortality (HR 1.17, 95% CI 0.98-1.41). When examining age during risk time as separate intervals, a significant association between mortality and AD was seen in the group of participants who died at the age of 65-80 years (HR 1.70, 95% CI 1.26-2.29). In the younger or older age interval, the association did not fully reach significance at the 95% level of confidence. Among confounding factors, smoking and physical activity were the strongest contributors. The association between smoking and mortality tended to be further increased in the group with anxiety disorders vs. the group without such disorders (HR 2.10, 95% CI 1.60-2.75 and HR 1.82, 95% CI 1.56-2.12, respectively).

Conclusions: This study suggests potential links between anxiety disorders, age, and mortality among women with 50 years of follow-up, but does not provide definitive conclusions due to the borderline significance of the results. No association between major depressive disorder and mortality was found.

Strengths and limitations of this study

For peer review only - http://bmjopen.bmj.com/site/about/guidelines.xhtml

- Strengths include a follow-up time of five decades, longitudinal design allows for a comprehensive examination of trends and associations over an extended period.
- The inclusion of a representative sample of middle-aged women in Gothenburg with high participation rates enhances the generalizability of the findings.
- The study considers various confounding factors such as smoking, alcohol consumption, physical activity, socioeconomic status, and educational level, which previous investigations often neglected.
- A psychiatric investigation based on DSM criteria rather than self-assessed symptoms at baseline ensures the accuracy of the disorders.
- Limitations are the relatively small sample size and an exclusively female population.

INTRODUCTION

Worldwide, mental disorders are among the top ten leading causes of morbidity. Major depressive disorder (MDD) and anxiety disorders (AD) are the most significant contributors to this, affecting more than half a billion people, and are overrepresented among women (1,2). The Swedish National Board of Health and Welfare states that persons with mental disorders have an increased risk of incidence as well as mortality in somatic disorders, compared to people without mental illness (3). However, the association between mental disorders and mortality is still debatable. In a Danish population-based cohort study it was seen that mental disorders were associated with a higher mortality rate, regardless of type of mental disorders, and to premature death, both for men and women (4). Also, comorbidity between mental disorder and general medical condition was associated to shorter life expectancy, compared to have one of the conditions only (5). Denollet et al. followed 5000 healthy Dutch women for ten years (6).They found that anxiety symptoms predicted premature all-cause and cardiovascular mortality (6). A later study distinguished between anxiety symptoms and anxiety disorders and followed persons for 11-19 years (7). No prediction of mortality, whether in women or men, was found (7). A Danish register study with nine years of follow-up showed that anxiety disorders significantly increased mortality risk, both in women and men (8). Cardiovascular disease and suicide are both potential contributors to increased mortality among people with MDD and AD (9-11). A variety of factors associated with poor health, such as hypertension, obesity and excess smoking, have also been linked to common mental disorders (12-16). The challenge remains to determine whether depression/anxiety at

For peer review only - http://bmjopen.bmj.com/site/about/guidelines.xhtml

baseline is associated with later smoking, or if smoking is associated with later depression/anxiety (17).

Two meta-analyses from 2015 and 2016 that studied the association between mortality and AD found conflicting results (18,19). One of the meta-analyses concluded that there was an association between mortality and AD, while the other found no such association. The diversity in results could be due to several reasons. Including psychiatric inpatients tended to increase the association (18), while studying a community sample and adjusting for comorbid AD/MDD had the opposite effect (19). Further, the identification of the disorders differed, with the majority based on medical records in one study (18) and self-reported symptoms in the other (19). However, the follow-up time was a median of ten years in one of the meta-analyses but less in the other, which limits the evaluation of the long-term effect. MDD, on the other hand, has repeatedly been associated with increased all-cause mortality, especially among men (20-22). In the Stirling County Study, Murphy et al. showed an association between depression and premature death among men (HR 2.6, 95% CI 1.4-4.9) in 10 years of follow-up. However, this was not seen among depressed women (HR 1.4, 95% CI 0.6-3.2) (23).

In 1968, the Prospective Population Study of Women in Gothenburg started, including middle-aged women (24). Because of its rather extensive content, the repeated follow-up exams and information regarding mortality during more than 50 years, it was possible to study the associations between disorders and mortality from middle age to the elderly. Given the contradictory results in previous research regarding AD and mortality, the aim was primarily to investigate a possible association between all-cause mortality and AD, secondarily MDD and comorbid AD/MDD, respectively. Another aim was to analyse the risk and health factors which influence such a possible association.

METHODS AND MATERIAL

The Population Study of Women in Gothenburg includes a representative sample of 1462 women aged 38, 46, 50, 54 and 60 years old who were invited to a medical examination in 1968-69. The participation rate was 90.1% (24). 899 participants were systematically sampled from the four youngest age groups according to birth date and invited for a psychiatric examination. Nighty-nine participants declined the invitation, and 22 participants were

For peer review only - http://bmjopen.bmj.com/site/about/guidelines.xhtml

excluded because of incomplete data due to language difficulties, psychosis or intellectual disability, leading to a total of 778 participants who fulfilled the psychiatric exam and were included in this study (see Flowchart in Appendix Figure 1). In this subsample, the participation rate was 89% of the invited subjects in the general population. Of those, 22 women were excluded from the present study because of incomplete data due to language difficulties, psychosis or intellectual disability. A comparison between non-participants and participants showed more single women among the non-participants. Still, no differences were found concerning mental disorders or the need for hospitalisation because of them (25,26). The psychiatric investigation was 1-2 hours long and was conducted using a comprehensive structured questionnaire.

Anxiety disorders and Major Depressive Disorder at baseline

A psychiatrist performed the psychiatric examination through a face-to-face interview (27). The findings from the psychiatric investigation were later revised by scrutinising items in the interview questionnaire relevant to a DSM-III MDD diagnosis (25). Cases of AD was identified from the dataset using algorithms based on the DSM-IV criteria. AD included social phobia, panic disorder, agoraphobia, generalised anxiety disorder (GAD) and obsessive-compulsive disorder (OCD). Identifying cases of posttraumatic stress disorder from items in the dataset was impossible. Specific phobias were excluded due to their generally limited clinical importance.

All-cause mortality rate

Information regarding participants' mortality was collected from the Swedish Cause of Death Register. The mortality rate was observed from the first examination date until the 31st of December 2019.

Statistics

Baseline characteristics were described using chi-square and independent t-test. The association between the baseline characteristics in the women with and without AD/MDD was measured by the Relative Risk (RR) and 95% confidence interval.

Poisson regression models were used to analyse associations with mortality, and the effect of assumed risk factors on mortality risk is reported as Hazard Ratio and 95% Confidence Interval (HR, 95% CI) .

For peer review only - http://bmjopen.bmj.com/site/about/guidelines.xhtml

The follow-up time of each individual is split into many short intervals of equal length. For each interval an event outcome variable is defined, coded 0 for not dead and 1 if not dead in that time interval. This structures survival data as a time-to-event dataset that will for all practical purposes give identical results whether analysed in a Cox model or a Poisson model. An advantage with Poisson models is that age and calendar time can be modelled as continuous risk factors, making it easy to test for interaction with regard to age or calendar time, that is, to test if the effect of a certain (assumed) risk factor varies by age or over calendar years. The hazard ratio (HR, CI 95%) measures the relative risk of death at any arbitrarily chosen time interval that, in the absence of interaction is assumed to be a constant. The risk time was measured from baseline to the time of death or 31st December 2019. The median follow-up time was 36.9 years (minimum 0.33, maximum 51.3 years). The total risk time was 27260 years and subdivided into quarters of a year (91.3 days).

When analysing the association between mortality and all AD and all MDD, covariates were sequentially added (Table 1). Still, each covariate was tested in a separate model with age as the only covariate, as small sample sizes limited the number of covariates that it would be meaningful to enter simultaneously (Table 2).

Statistical significance was accepted at $p < 0.05$ in all analyses except tests of interaction, which was accepted at $p < 0.10$. SPSS version 29 and Stata version 17 were used to perform statistical analysis.

Potential confounders

To reduce the effect of potential confounders, the following covariates, with known influence on mortality, were used: age (in years), education (over or under six years), economic status (defined as annual income divided by number of family members), alcohol consumption (from never to everyday use of wine, beer and liquor, separately, and "regularly" is defined as several days per week or more), smoking (current or non-current), physical activity ("sedentary leisure time" is defined as <4 hours/week)(28) and BMI (weight in kilogram divided with the square of height in meters). Lower educational attainment and income levels have been associated with higher mortality rates (29) and marital status can influence mortality, with married individuals experiencing lower mortality rates compared to those who are unmarried (30).

All covariates except age were dichotomised in the regression models. These data were collected in interviews or measured at baseline.

For peer review only - http://bmjopen.bmj.com/site/about/guidelines.xhtml

RESULTS

AD, MDD and comorbid AD/MDD

One hundred sixty participants (20.6%) had one or more AD, and 57 (7.3%) had MDD in the psychiatric exam 1968-69. Of these, 25 participants (3.2%) had comorbid AD/MDD. The numbers of those with AD solely and MDD solely are presented in Table 1.

The distribution of disorders among AD was GAD n=71 (9.1%), agoraphobia n=67 (8.6%), panic disorder n=58 (7.4%), social phobia n=51 (6.6%) and obsessive-compulsive disorder (OCD) n=28 (3.6%). Of the 135 participants with AD, 85 had one disorder, 48 had two, 17 had three, 7 had four, and 3 had all five disorders.

Baseline characteristics

Baseline characteristics are shown in Table 1. Participants with AD solely (i.e. excluding participants with comorbid AD/MDD) did, to a greater extent, smoke and consume more alcohol (beer) than those without any disorder. Participants with comorbid AD/MDD had significantly more sedentary leisure time and lower economic status. Participants with MDD were, to a lesser extent, married.

Table 1. Baseline characteristics of the different groups of women with AD solely, MDD solely, comorbid AD/MDD and no AD or MDD, at the examination year 1968-69.

**Characteristics, Mean (SD)**	AD solely n=135	MDD solely n=32	Comorbid AD/MDD n=25	No AD or MDD n=586
Age (years)	47.6 (4.4)	47.8 (5.1)	47.2 (5.3)	47.9 (4.5)
Economic status[1], 1000 SEK	16.2 (8.5)	16.6 (12.2)	**12.0 (4.4)** *	16.1 (9.8)
Number of cigarettes per day among smokers	10.5 (6.0)	**14.1 (7.9)** *	11.2 (8.3)	10.2 (6.0)
BMI kg/m2	24.4 (4.3)	23.7 (2.6)	24.2 (3.4)	24.1 (3.7)
**Characteristics, Prevalence (%)**				
Education six years or more, n (%)	32 (23.7)	6 (18.8)	3 (12.0)	153 (26.1)
Marital status, married	102 (75.6)	**19 (59.4)** *	22 (88)	483 (82.4)
Physical activity Sedentary leisure time[2], n (%)	27 (20.6)	4 (12.5)	**8 (32.0)** *	95 (16.2)
Smoking, n (%)	**71 (52.6)** **	16 (50.0)	13 (52.0)	226 (38.8)

For peer review only - http://bmjopen.bmj.com/site/about/guidelines.xhtml

Economic status (in 10% lowest income category) n (%)	13 (9.6)	2 (6.3)	1 (4.2)	55 (9.5)
Regular alcohol use, n (%)	**49 (36.2) ****			
Beer	8 (5.9)	5 (15.6)	5 (20)	162 (27.6)
Wine	1 (0.7)	0 (0)	0 (0)	33 (5.6)
Spirits		0 (0)	0 (0)	8 (1.4)
BMI >30	13 (9.6)	1 (3.1)	1 (4.0)	44 (7.5.)
BMI <19	8 (5.9)	1 (3.1)	2 (8.0)	28 (4.8)

* $p < 0.05$ ** $p < 0.01$
[1]Annual income divided by number of family members
[2]<4 hours/week. Values in the cell 'Physical activity Sedentary leisure time' and 'AD solely n=135' mean that 27 women belong to the sedentary category and they comprise 20.6% of those in the AD solely group (5 are missing data on physical activity). The values show the number of persons with a sedentary lifestyle and their proportion in each AD/MDD category.
The significance is tested for the chosen group compared to the group with neither AD nor MDD.

peer review only

Smoking, beer consumption and physical activity (measured at baseline)

Having AD without MMD significantly increased the relative risk of being a smoker (RR 1.38, CI 1.13-1.67, p<0.01) and regularly consuming beer (RR 1.35, CI 1.05-1.75, p<0.05), compared to not having any of the disorders. Having MDD without AD increased the relative risk of being a heavy smoker (>13 cigarettes/day) (RR 2.23, CI 1.20-4.33, p<0.05). Having comorbid AD/MDD significantly increased the relative risk of having a sedentary leisure time (RR 1.97, CI 1.08-3.59, p=0.05).

All cases of AD and MDD and all-cause mortality rates

Of the total 778 participants, 720 died before the 1st of January 2020. A multivariate regression survival model was used to examine the associations between all cases of AD, all cases of MDD and mortality (Table 2). In the full model, there was no association between AD and mortality. In a corresponding analysis of all cases of MDD, there was a significant association when adjusted for age.

Table 2. Associations between all cases of AD, all cases of MDD and mortality rate in different adjustment models.

	**AD n=160**	**MDD n=57**
**Covariates**	HR (95% CI)	
Age	**1.25 (1.04-1.49) ***	**1.21 (1.11-1.12) ***

For peer review only - http://bmjopen.bmj.com/site/about/guidelines.xhtml

Age, smoking, physical activity, and alcohol use (beer)	**1.20 (1.00-1.44) ***	1.18 (0.90-1.56)
All of the above, economic status, married, education, BMI>30 and BMI<19	1.17 (0.98-1.41)	1.19 (0.90-1.57)
All of the above and depression	1.16 (0.96-1.39)	-
All of the above and anxiety	-	1.15 (0.86-1.52)
Bold figures, statistically significant		

When restricting the analysis (full model including all AD and all MDD) to deaths occurring before the age of 80, AD became a significant risk factor (HR 1.49, CI 1.13-1.98, p<0.01). This association was even stronger in the age range of 65-80 years (HR 1.70, CI 1.26-2.29, p<0.001).

To test the significance of interaction for the effect of AD on mortality in different age intervals, one model with three separate AD-effect parameters was calculated for the age groups 38-64, 65-79 and 80-101. This model was compared to a base model assuming constant AD-effect over the ages. The difference in overall model Chi-square between the two Poisson regression models was 8.2 with df=2 and p=0.02.

An age-adjusted model with only two AD-effect parameters, the first for age 65-79 and the second for the younger and older age intervals pooled, yielded Chi-square= 9.3, df=1 and p=0.002 compared to the base model. A fully adjusted model yielded Chi-square=7.94, df=1 and p=0.005.

When analyzing the relationship between all-cause mortality and MDD, no significant associations were found. However, it's worth noting that the Incidence Rate Ratio (IRR) exhibited a higher magnitude compared to AD, albeit with somewhat less precision.

AD solely, MDD solely, comorbid AD/MDD and all-cause mortality rates

When assessing single risk factors (with age included), a significant association with mortality was found among women with solely AD who smoked. A similar trend was noted in cases of comorbid AD/MDD, as seen in Table 3.

Table 3. Associations between single covariates and the mortality rate in the total sample and the groups with AD solely, MDD solely, and comorbid AD/ADD. All models include age.

	Total sample n=778	AD solely n=135	MDD solely n=32	Comorbid AD/MDD n=25

For peer review only - http://bmjopen.bmj.com/site/about/guidelines.xhtml

Covariate	HR (95% CI)	HR (95% CI)	HR (95% CI)	HR (95% CI)
1. Age	**1.13 (1.12-1.14)** ***	**1.13 (1.11-1.15)** ***	**1.14 (1.09-1.19)** ***	**1.17 (1.11-1.23)** ***
2. Education ≥ 6 years	0.88 (0.74-1.05)	1.00 (0.62-1.60)	1.20 (0.46-3.19)	0.30 (0.04-2.13)
3. Physical activity: Regular exercise	**0.73 (0.63-0.85)** ***	0.71 (0.49-1.04)	0.79 (0.38-1.66)	1.02 (0.40-2.63)
4. Smoking	**1.82 (1.56-2.13)** ***	**1.86 (1.24-2.79)** **	2.12 (0.95-4.72)	**5.18 (1.57-17.14)** **
5. Beer consumption	0.93 (0.84-1.03)	0.83 (0.66-1.05)	0.70 (0.38-1.29)	1.85 (0.76-4.49)
6. BMI<19	**1.66 (1.20-2.31)** ***	2.13 (0.98-4.62)	1.57 (0.17-14.07)	2.38 (0.19-29.8)
7. BMI>30	1.31 (1.00-1.72)	0.88 (0.47-1.67)	2.07 (0.25-16.88)	0.60 (0.07-4.86)
8. Married	1.00 (0.84-1.22)	0.97 (0.62-1.50)	1.29 (0.54-3.07)	1.08 (0.30-3.97)
9. Economic status (lowest decentile)	**1.37 (1.07-1.77)** *	1.43(0.82-2.49)	0.69 (0.22-2.23)	0.37 (0.05-2.86)

In a regression analysis, when adjusted for age, the increased mortality risk associated with smoking did increase further among those having AD, compared to not having AD, HR 2.28 and 1.71, but with overlapping 95% CIs, 1.64-3.17 and 1.44-2.02, respectively, p= 0.096. The corresponding difference in mortality risk associated with regular physical activity was negligible, with HR 0.77 (95% CI = 0.57-1.03) and HR 0.73 (95% CI = 0.62-0.86), p= 0.95.

For peer review only - http://bmjopen.bmj.com/site/about/guidelines.xhtml

DISCUSSION

Main findings

In this 50-year study of middle-aged women, several significant findings emerged. Women with solely AD were more likely to be smokers and beer consumers compared to those without AD or MDD. Notably, women with AD (all cases) exhibited a significantly increased mortality rate when considering age, smoking, physical activity, and beer consumption as covariates in the model. However, this association did not reach statistical significance in the full model. Conversely, women with MDD (all cases) displayed a non-significant trend toward an increased mortality rate in both models. When analyzing deaths specifically within the age range of 65-80 years, a strong and significant association was found between AD and increased mortality rate in. Intriguingly, no such associations were identified for the age intervals of 38-64 and 80-101, indicating a significant interaction effect for AD-Age-Mortality. Notably, no analogous pattern was observed concerning the interaction between MDD-Age-Mortality.

Comparison with other studies

The lack of significant overall association between MDD and all-cause mortality is in contrast to previous findings (20-22), with some exceptions. Holwerda et al. did find an association, but only among elderly men (22). Eaton et al., on the other hand, did not find any association, and the lack of this is suggested to be primarily due to a long follow-up time (27 years). Symptoms of MDD could be disguised signs of poor health. In the short term, this could lead to an increase in mortality risk that later would subside (31). However, if this were applicable in our study, it would have been revealed in our sample when analysing time intervals, which was not the case.

Our sample of women with MDD may have been too small, which may explain the lack of significant findings in this group. It is notable, however, that in most of our analyses of MDD and mortality, the hazard ratios were similar to those in the analyses of AD. For example, in the 80-101 age range the hazard ratio for MMD was 1.39 (95% CI 0.98-1.98).

The finding of a reduced association between AD and mortality rate when the full model was applied is consistent with previous research (19,21,31). The increased mortality rate among women with AD in the age range 65-80 is not readily comparable to earlier studies due to the difference in follow-up time. One interpretation of this finding could be that AD is associated

For peer review only - http://bmjopen.bmj.com/site/about/guidelines.xhtml

with mortality only in ages >65 years. This contradicts previous results, where AD in older women has not been associated with increased mortality but concurs with our finding of no association after age 80 (22,32). Another interpretation is that AD that lasts for an extended period of time, increases vulnerability and may impede the ability of individuals to manage their health and access necessary medical care, which can further contribute to an increased morbidity and mortality risk.

Smoking is an established risk factor for mortality. The association between smoking and mortality tended to increase further in the presence of AD and comorbid AD/MDD. A different pattern in smoking habits could explain this. We found that smoking was more common among women with AD. Another study reported that persons with AD, to a lesser extent, cease daily smoking. If they do, the duration of smoking is longer (33). Our study did not investigate the exposure time. Still, if our sample is comparable to that study, and the women had a longer duration of smoking, this could be one possible contributing cause to the late increase in mortality.

A meta-analysis of smoking and mortality found that the mortality rate decreased among female smokers after 75 or 80 years (34). This is consistent with our findings of reduced mortality after 80 years among women with AD, where 53% were smoking at baseline. One possible explanation is that after the age of 80 years, subjects susceptible to harmful smoking effects may have died already. Another possible reason might be that after the age of 80 years, other causes of death increase and compete with those related to smoking and AD.

Strengths and weaknesses

This study exhibits several strengths, including its extended five-decade longitudinal design, a representative sample of middle-aged women in Gothenburg with high participation rates, and a comprehensive consideration of various confounding factors. Importantly, prior investigations reporting increased mortality rates in anxiety disorders did not control for covariates like smoking, alcohol consumption, physical activity, socioeconomic status, or educational level, which could have influenced their findings (4,5,8).

Also, many studies investigating AD and MDD have used various self-administrated questionnaires, measuring symptomatology rather than the frequency of a disorder. This could

For peer review only - http://bmjopen.bmj.com/site/about/guidelines.xhtml

potentially over- or underdiagnose the psychopathology, and the reported symptoms could originate from a somatic condition. In this study, however, the disorder was assessed by a psychiatrist, making the results more accurate. Furthermore, studies tend to investigate associations either among persons with AD or MDD, which could be misleading due to the high comorbidity. In contrast, in the current study the participants with comorbid AD/MDD were examined as a separate group.

The study has some limitations. Firstly, the research sample was relatively small, especially regarding the group having MDD and comorbid AD/MDD. While there is a potential for complex causal mechanisms linking depression status, lifestyle factors such as smoking and alcohol consumption, and mortality risks, our study is primarily focused on examining associations and variations. Introducing complex models would not enhance our analysis, as our dataset lacks the necessary scope to support such an approach. Another limitation was that the study sample only included women. Furthermore, somatic diseases among the participants were not taken into account. It is possible that patients with AD could have a higher rate of somatic disease, which in turn could affect the mortality rate. Lastly, it's important to acknowledge that the decision made at the outset to confine the evaluation of baseline risk factors and their outcomes, as well as to assess the point prevalence of AD and MDD (not the lifetime prevalence), represents a limitation in this study. However, it's worth noting that this limitation may not necessarily introduce biases, especially when taking into account the specific research focus of our study. However, the onset of AD is, above all, in early adolescence to young adulthood and often has a long duration, which is why we can consider that the participants who meet the criterion for AD in middle age have had the disorder for a long time. Correspondingly, most of those with the preponderance to incur the condition should have received it by the time of the baseline examination. The same is true for MDD, which has a median onset in young adulthood (35).

The significance of the study, unanswered questions and future research

This study adds to the previous findings showing that AD is not convincingly associated with mortality over the entire age interval studied. The same applies to comorbid AD/MDD. The MDD group was small, however, and the results concerning MDD and mortality are therefore inconclusive. The increase in mortality among those with AD aged 65-80 years is a novel finding that needs to be confirmed in future studies. Considering the high prevalence of comorbid AD/MDD cases, future studies on the association between mortality and AD or

For peer review only - http://bmjopen.bmj.com/site/about/guidelines.xhtml

MDD should include both disorders. Future research should also focus on effective methods to promote a healthier lifestyle among persons with mental disorders especially regarding cessation of smoking and reduction of heavy alcohol consumption to counteract the increased likelihood of developing comorbid medical conditions such as cardiovascular disease.

Funding

This work was supported by Grants from the Swedish Research Council for Health, Working Life and Welfare—FORTE 2007-1958 and grants from the Swedish State under the agreement between the Swedish Government and the county council, the ALF agreement GBG-68771. This research received no specific grant from any public, commercial or not-for-profit sector funding agency.

Competing interests

None declared.

Each author's contribution

The study was conducted as part of AvBs intern research project. DH, TH and CB supervised the work. TH was responsible for the AD and MDD diagnostic procedures. AvB analysed the data and prepared the draft manuscript with the support of all the other authors, DH, TH and CB. VS devised the statistical analysis and contributed to the interpretation of it. All the authors approved the final manuscript and revision.

Data sharing statement

Data are available on reasonable request. All data relevant to the study are included in the article.

Ethics Approval Statement

The Regional Ethical Committee in Gothenburg approved the study (258-16 T853-16). The research was conducted following the World Medical Association Declaration of Helsinki, and all participants provided written informed consent. The manuscript does not contain personal and/or medical information about an identifiable individual. The manuscript is sufficiently anonymised in line with BMJ Open anonymisation policy and checklist. All data are analysed on group level.

For peer review only - http://bmjopen.bmj.com/site/about/guidelines.xhtml

For peer review only

REFERENCES

1. GBD 2019 Mental Disorders Collaborators. Global, regional, and national burden of 12 mental disorders in 204 countries and territories, 1990-2019: a systematic analysis for the Global Burden of Disease Study 2019. *Lancet Psychiatry*. 2022;9:137-150. doi:10.1016/S2215-0366(21)00395-3
2. Dattani S, Ritchie H. Roser M. "Mental Health". Published online at OurWorldInData.org. 2021.Retrieved from: 'https://ourworldindata.org/mental-health' [Online Resource] May 9, 2023
3. National Board of Health and Welfare, Sweden. Evaluation of care at depression and anxiety disorders. National guidelines – Evaluation Main report with areas for improvement (Socialstyrelsen. Utvärdering av vård vid depression och ångestsyndrom Nationella riktlinjer – Utvärdering. Huvudrapport med förbättringsområden) https://www.socialstyrelsen.se/globalassets/sharepointdokument/artikelkatalog/nationella-riktlinjer/2019-5-12.pdf. 2019.
4. Plana-Ripoll O, Pedersen CB, Agerbo E, Holtz Y, Erlangsen A, Canudas-Romo V, et al. A comprehensive analysis of mortality-related health metrics associated with mental disorders: a nationwide, register-based cohort study. Lancet. 2019;394(10211):1827-35.
5. Momen NC, Plana-Ripoll O, Agerbo E, Christensen MK, Iburg KM, Laursen TM, et al. Mortality Associated With Mental Disorders and Comorbid General Medical Conditions. JAMA Psychiatry. 2022;79(5):444-53.

6. Denollet J, Maas K, Knottnerus A, Keyzer JJ, Pop VJ. Anxiety predicted premature all-cause and cardiovascular death in a 10-year follow-up of middle-aged women. *Journal of clinical epidemiology*. 2009;62:452-456. doi:10.1016/j.jclinepi.2008.08.006
7. Saeed Mirza S, Ikram MA, Hofman A, Tiemeier H. Anxiety does not predict mortality. A population-based study. *World Psychiatry*. 2015;14:103-104. doi:10.1002/wps.20192
8. Meier SM, Mattheisen M, Mors O, Mortensen PB, Laursen TM, Penninx BW. Increased mortality among people with anxiety disorders: total population study. *Br J Psychiatry*. 2016;209:216-221. doi:10.1192/bjp.bp.115.171975
9. Roest AM, Martens EJ, de Jonge P, Denollet J. Anxiety and risk of incident coronary heart disease: a meta-analysis. *J Am Coll Cardiol*. 2010;56:38-46. doi:10.1016/j.jacc.2010.03.034
10. Van der Kooy K, van Hout H, Marwijk H, Marten H, Stehouwer C, Beekman A. Depression and the risk for cardiovascular diseases: systematic review and meta analysis. *Int J Geriatr Psychiatry*. 2007;22:613-626. doi:10.1002/gps.1723
11. Moitra M, Santomauro D, Degenhardt L, et al. Estimating the risk of suicide associated with mental disorders: A systematic review and meta-regression analysis. *J Psychiatr Res*. 2021;137:242-249. doi:10.1016/j.jpsychires.2021.02.053
12. Luppino FS, de Wit LM, Bouvy PF, et al. Overweight, obesity, and depression: a systematic review and meta-analysis of longitudinal studies. *Arch Gen Psychiat*. 2010;67:220-9. doi:10.1001/archgenpsychiatry.2010.2
13. Konttinen H, van Strien T, Mannisto S, Jousilahti P, Haukkala A. Depression, emotional eating and long-term weight changes: a population-based prospective study. *Int J Behav Nutr Phys Act*. 2019;16:28. doi:10.1186/s12966-019-0791-8

For peer review only - http://bmjopen.bmj.com/site/about/guidelines.xhtml

14. Brumpton B, Langhammer A, Romundstad P, Chen Y, Mai XM. The associations of anxiety and depression symptoms with weight change and incident obesity: The HUNT Study. *Int J Obes (Lond)*. 2013;37:1268-74. doi:10.1038/ijo.2012.204
15. Lim LF, Solmi M, Cortese S. Association between anxiety and hypertension in adults: A systematic review and meta-analysis. *Neurosci Biobehav Rev*. 2021;131:96-119. doi:10.1016/j.neubiorev.2021.08.031
16. Weinberger AH, Chaiton MO, Zhu J, Wall MM, Hasin DS, Goodwin RD. Trends in the Prevalence of Current, Daily, and Nondaily Cigarette Smoking and Quit Ratios by Depression Status in the U.S.: 2005-2017. *Am J Prev Med*. 2020;58:691-698. doi:10.1016/j.amepre.2019.12.023
17. Fluharty M, Taylor AE, Grabski M, Munafo MR. The Association of Cigarette Smoking With Depression and Anxiety: A Systematic Review. *Nicotine Tob Res*. 2017;19:3-13. doi:10.1093/ntr/ntw140
18. Walker ER, McGee RE, Druss BG. Mortality in mental disorders and global disease burden implications: a systematic review and meta-analysis. *JAMA Psychiatry*. 2015;72:334-41. doi:10.1001/jamapsychiatry.2014.2502
19. Miloyan B, Bulley A, Bandeen-Roche K, Eaton WW, Goncalves-Bradley DC. Anxiety disorders and all-cause mortality: systematic review and meta-analysis. *Soc Psychiatry Psychiatr Epidemiol*. 2016;51:1467-1475. doi:10.1007/s00127-016-1284-6
20. van Dijk MR, Utens EM, Dulfer K, et al. Depression and anxiety symptoms as predictors of mortality in PCI patients at 10 years of follow-up. *Eur J Prev Cardiol*. 2016;23:552-558. doi:10.1177/2047487315571889
21. Mykletun A, Bjerkeset O, Overland S, Prince M, Dewey M, Stewart R. Levels of anxiety and depression as predictors of mortality: the HUNT study. *Br J Psychiatry*. 2009;195:118-125. doi:10.1192/bjp.bp.108.054866
22. Holwerda TJ, Schoevers RA, Dekker J, Deeg DJ, Jonker C, Beekman AT. The relationship between generalized anxiety disorder, depression and mortality in old age. *Int J Geriatr Psychiatry*. 2007;22:241-249. doi:10.1002/gps.1669
23. Murphy JM, Gilman SE, Lesage A, et al. Time trends in mortality associated with depression: findings from the Stirling County study. *Can J Psychiatry*. 2010;55:776-783. doi:10.1177/070674371005501205
24. Bengtsson C, Blohme G, Hallberg L, et al. The study of women in Gothenburg 1968-1969-a population study. General design, purpose and sampling results. *Acta medica Scandinavica*. 1973;193:311-318.
25. Hällström T. Point prevalence of major depressive disorder in a Swedish urban female population. *Acta Psychiatr Scand*. 1984;69:52-9. doi:10.1111/j.1600-0447.1984.tb04516.x
26. Hällström T. *Mental disorder and sexuality in the climacteric : a study in psychiatric epidemiology*. Scandinavian University Books, Esselte Studium, Gothenburg. Diss. Gothenburg : Univ.; 1973.
27. Åsberg M, Montgomery SA, Perris C, Schalling D, Sedvall G. A comprehensive psychopathological rating scale. *Acta Psychiatr Scand Suppl*. 1978:5-27. doi:10.1111/j.1600-0447.1978.tb02357.x
28. Saltin B, Grimby G. Physiological analysis of middle-aged and old former athletes. Comparison with still active athletes of the same ages. *Circulation*. 1968;38:1104-1115. doi:10.1161/01.cir.38.6.1104

For peer review only

For peer review only - http://bmjopen.bmj.com/site/about/guidelines.xhtml

29. Backlund E, Sorlie PD, Johnson NJ. The shape of the relationship between income and mortality in the United States. Evidence from the National Longitudinal Mortality Study. Annals of epidemiology. 1996;6(1):12-20; discussion 1-2.
30. Manzoli L, Villari P, G MP, Boccia A. Marital status and mortality in the elderly: a systematic review and meta-analysis. Social science & medicine (1982). 2007;64(1):77-94.

31. Eaton WW, Roth KB, Bruce M, et al. The relationship of mental and behavioral disorders to all-cause mortality in a 27-year follow-up of 4 epidemiologic catchment area samples. *American journal of epidemiology*. 2013;178:1366-77. doi:10.1093/aje/kwt219
32. van Hout HP, Beekman AT, de Beurs E, et al. Anxiety and the risk of death in older men and women. *Br J Psychiatry*. 2004;185:399-404. doi:10.1192/bjp.185.5.399
33. Lawrence D, Considine J, Mitrou F, Zubrick SR. Anxiety disorders and cigarette smoking: Results from the Australian Survey of Mental Health and Wellbeing. *Aust N Z J Psychiatry*. 2010;44:520-527. doi:10.3109/00048670903571580
34. Gellert C, Schottker B, Brenner H. Smoking and all-cause mortality in older people: systematic review and meta-analysis. *Arch Intern Med*. 2012;172:837-844. doi:10.1001/archinternmed.2012.1397
35. Solmi M, Radua J, Olivola M, et al. Age at onset of mental disorders worldwide: large-scale meta-analysis of 192 epidemiological studies. *Mol Psychiatry*. 2022;27:281-295. doi:10.1038/s41380-021-01161-7

For peer review only

Appendix Figure 1

Flowchart of participants in Population Study of Women in Gothenburg in 1968-69

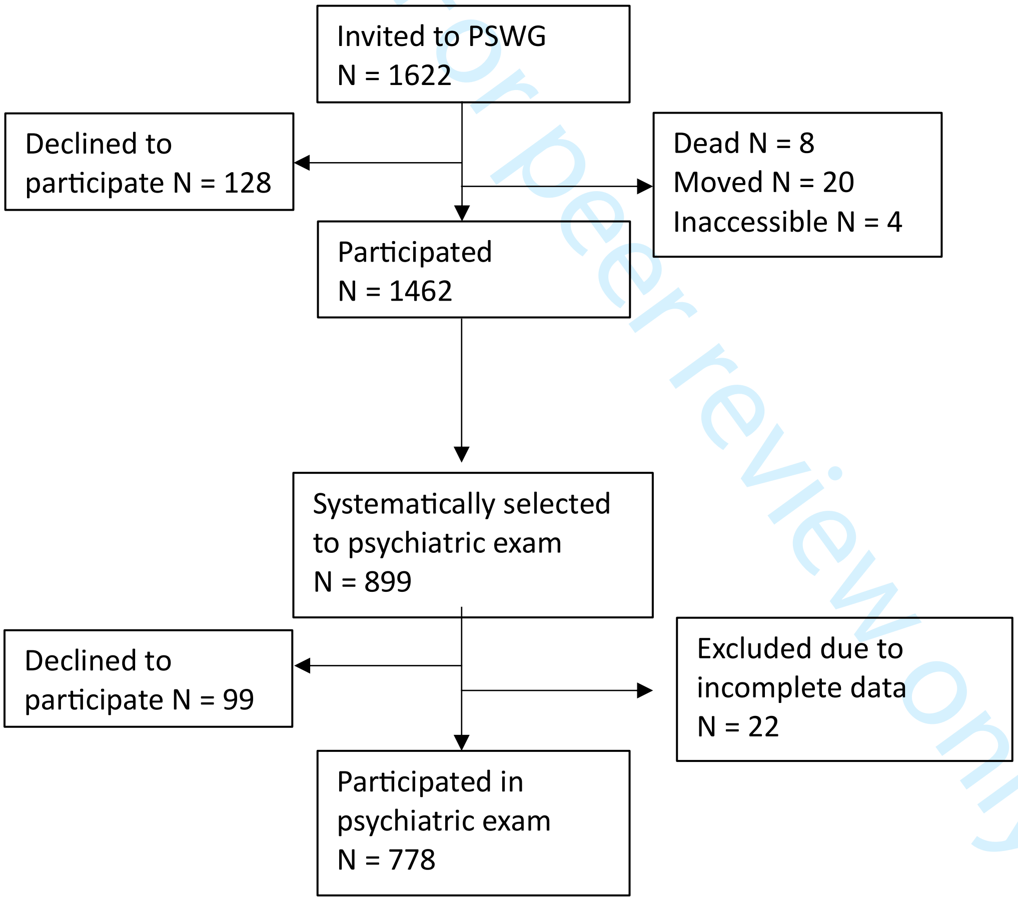

For peer review only

Reporting checklist for cohort study.

Based on the STROBE cohort guidelines.

Instructions to authors

Complete this checklist by entering the page numbers from your manuscript where readers will find each of the items listed below.

Your article may not currently address all the items on the checklist. Please modify your text to include the missing information. If you are certain that an item does not apply, please write "n/a" and provide a short explanation.

Upload your completed checklist as an extra file when you submit to a journal.

In your methods section, say that you used the STROBE cohortreporting guidelines, and cite them as:

von Elm E, Altman DG, Egger M, Pocock SJ, Gotzsche PC, Vandenbroucke JP. The Strengthening the Reporting of Observational Studies in Epidemiology (STROBE) Statement: guidelines for reporting observational studies.

		Reporting Item	Page Number
**Title and abstract**			
Title	#1a	Indicate the study’s design with a commonly used term in the title or the abstract	1
Abstract	#1b	Provide in the abstract an informative and balanced summary of what was done and what was found	2
**Introduction**			
Background / rationale	#2	Explain the scientific background and rationale for the investigation being reported	3
Objectives	#3	State specific objectives, including any prespecified hypotheses	4
**Methods**			
Study design	#4	Present key elements of study design early in the paper	4

For peer review only

Setting	#5	Describe the setting, locations, and relevant dates, including periods of recruitment, exposure, follow-up, and data collection	4
Eligibility criteria	#6a	Give the eligibility criteria, and the sources and methods of selection of participants. Describe methods of follow-up.	4
Eligibility criteria	#6b	For matched studies, give matching criteria and number of exposed and unexposed	n/a
Variables	#7	Clearly define all outcomes, exposures, predictors, potential confounders, and effect modifiers. Give diagnostic criteria, if applicable	5
Data sources / measurement	#8	For each variable of interest give sources of data and details of methods of assessment (measurement). Describe comparability of assessment methods if there is more than one group. Give information separately for for exposed and unexposed groups if applicable.	5
Bias	#9	Describe any efforts to address potential sources of bias	n/a
Study size	#10	Explain how the study size was arrived at	4
Quantitative variables	#11	Explain how quantitative variables were handled in the analyses. If applicable, describe which groupings were chosen, and why	5
Statistical methods	#12a	Describe all statistical methods, including those used to control for confounding	
5			
Statistical methods	#12b	Describe any methods used to examine subgroups and interactions	5
Statistical methods	#12c	Explain how missing data were addressed	5
Statistical methods	#12d	If applicable, explain how loss to follow-up was addressed	5
Statistical methods	#12e	Describe any sensitivity analyses	
5			
**Results**			

For peer review only

Participants	#13a	Report numbers of individuals at each stage of study—eg numbers potentially eligible, examined for eligibility, confirmed eligible, included in the study, completing follow-up, and analysed. Give information separately for for exposed and unexposed groups if applicable.	6
Participants	#13b	Give reasons for non-participation at each stage	5
Participants	#13c	Consider use of a flow diagram	
n/a			
Descriptive data	#14a	Give characteristics of study participants (eg demographic, clinical, social) and information on exposures and potential confounders. Give information separately for exposed and unexposed groups if applicable.	6
Descriptive data	#14b	Indicate number of participants with missing data for each variable of interest	
n/a			
Descriptive data	#14c	Summarise follow-up time (eg, average and total amount)	
6			
Outcome data	#15	Report numbers of outcome events or summary measures over time. Give information separately for exposed and unexposed groups if applicable.	
7			
Main results	#16a	Give unadjusted estimates and, if applicable, confounder-adjusted estimates and their precision (eg, 95% confidence interval). Make clear which confounders were adjusted for and why they were included	8
Main results	#16b	Report category boundaries when continuous variables were categorized	n/a
Main results	#16c	If relevant, consider translating estimates of relative risk into absolute risk for a meaningful time period	
8			

For peer review only

Other analyses	#17	Report other analyses done—eg analyses of subgroups and interactions, and sensitivity analyses	n/a
**Discussion**			
Key results	#18	Summarise key results with reference to study objectives	10
Limitations	#19	Discuss limitations of the study, taking into account sources of potential bias or imprecision. Discuss both direction and magnitude of any potential bias.	12
Interpretation	#20	Give a cautious overall interpretation considering objectives, limitations, multiplicity of analyses, results from similar studies, and other relevant evidence.	12
Generalisability	#21	Discuss the generalisability (external validity) of the study results	12
**Other Information**			
Funding	#22	Give the source of funding and the role of the funders for the present study and, if applicable, for the original study on which the present article is based	12

The STROBE checklist is distributed under the terms of the Creative Commons Attribution License CC-BY. This checklist was completed on 09. May 2023 using https://www.goodreports.org/, a tool made by the EQUATOR Network in collaboration with Penelope.ai

For peer review only

Association between Anxiety and Depression and all-cause Mortality: a 50-year follow-up of the Population Study of Women in Gothenburg, Sweden

Journal:	*BMJ Open*
Manuscript ID	bmjopen-2023-075471.R2
Article Type:	Original research
Date Submitted by the Author:	29-Oct-2023

Complete List of Authors:	von Below, Amanda; University of Gothenburg Sahlgrenska Academy, Primary Health Care/School of Public Health and Community Medicine<br>Hällström, Tore; Institute of Neuroscience and Physiology, Sahlgrenska Academy, Department of Psychiatry and Neurochemistry, Gothenburg University<br>Sundh, Valter; University of Gothenburg Sahlgrenska Academy, Primary Health Care/School of Public Health and Community Medicine<br>Björkelund, Cecilia; University of Gothenburg Sahlgrenska Academy, Primary Health Care/School of Public Health and Community Medicine<br>Hange, Dominique; University of Gothenburg Sahlgrenska Academy, Primary Health Care/School of Public Health and Community Medicine

**Primary Subject Heading**:	General practice / Family practice
Secondary Subject Heading:	Mental health
Keywords:	Anxiety disorders < PSYCHIATRY, Depression & mood disorders < PSYCHIATRY, Primary Care < Primary Health Care, Adult psychiatry < PSYCHIATRY, PUBLIC HEALTH

SCHOLARONE™
Manuscripts

I, the Submitting Author has the right to grant and does grant on behalf of all authors of the Work (as defined in the below author licence), an exclusive licence and/or a non-exclusive licence for contributions from authors who are: i) UK Crown employees; ii) where BMJ has agreed a CC-BY licence shall apply, and/or iii) in accordance with the terms applicable for US Federal Government officers or employees acting as part of their official duties; on a worldwide, perpetual, irrevocable, royalty-free basis to BMJ Publishing Group Ltd ("BMJ") its licensees and where the relevant Journal is co-owned by BMJ to the co-owners of the Journal, to publish the Work in this journal and any other BMJ products and to exploit all rights, as set out in our licence.

The Submitting Author accepts and understands that any supply made under these terms is made by BMJ to the Submitting Author unless you are acting as an employee on behalf of your employer or a postgraduate student of an affiliated institution which is paying any applicable article publishing charge ("APC") for Open Access articles. Where the Submitting Author wishes to make the Work available on an Open Access basis (and intends to pay the relevant APC), the terms of reuse of such Open Access shall be governed by a Creative Commons licence – details of these licences and which Creative Commons licence will apply to this Work are set out in our licence referred to above.

Other than as permitted in any relevant BMJ Author's Self Archiving Policies, I confirm this Work has not been accepted for publication elsewhere, is not being considered for publication elsewhere and does not duplicate material already published. I confirm all authors consent to publication of this Work and authorise the granting of this licence.

For peer review only

Association between Anxiety and Depression and all-cause Mortality: a 50-year follow-up of the Population Study of Women in Gothenburg, Sweden

Amanda von Below[1], Tore Hällström[2], Valter Sundh[1], Cecilia Björkelund[1,3], Dominique Hange[1,3]

[1]*Primary Health Care, School of Public Health and Community Medicine, Institute of Medicine, Sahlgrenska Academy, University of Gothenburg, Sweden*

[2]*Neuropsychiatric Epidemiology Unit, Department of Psychiatry and Neurochemistry, Institute of Neuroscience and Physiology, Sahlgrenska Academy, Centre for Aging and Health (AGECAP), University of Gothenburg, Sweden*

[3]*Research, Education, Development & Innovation, Primary Health Care, Region Västra Götaland, Sweden*

Corresponding author

Dominique Hange

Department of Public Health and Community Medicine/Primary Health Care

PO Box 453,

SE-405 30 Gothenburg, Sweden

Phone: +46-31-773 68 49 Fax: +46-31-778 17 04

E-Mail: dominique.hange@allmed.gu.se

For peer review only - http://bmjopen.bmj.com/site/about/guidelines.xhtml

Abstract

Objectives: To examine the association between anxiety disorders and/or major depression disorder and all-cause mortality in a 50-year perspective and to examine specific risk and health factors that may influence such an association.

Design: Observational population study, 1968-2019.

Setting: The Population Study of Women in Gothenburg, Sweden (PSWG).

Participants: In 1968-69, 899 (out of in total of 1462) women from PSWG were selected according to date of birth for a psychiatric investigation, including diagnostic evaluation. Eight hundred (89%) accepted. Twenty-two women were excluded. Of the 778 included, 135 participants (17.4 %) had solely anxiety disorders (AD), 32 (4.1%) had solely major depressive disorder (MDD) and 25 (3.2%) had comorbid AD/MDD.

Primary and secondary outcome measures: Associations between anxiety disorders, major depressive disorder, comorbid AD/MDD and all-cause mortality with adjustments for potential confounding factors. Differences between the groups concerning health and risk factors and their association with mortality.

Results: In a fully-adjusted model, anxiety disorders were non-significantly associated with all-cause mortality (HR 1.17, 95% CI 0.98-1.41). When examining age during risk time as separate intervals, a significant association between mortality and AD was seen in the group of participants who died at the age of 65-80 years (HR 1.70, 95% CI 1.26-2.29). In the younger or older age interval, the association did not reach significance at the 95% level of confidence. Among confounding factors, smoking and physical activity were the strongest contributors. The association between smoking and mortality tended to be further increased in the group with anxiety disorders vs. the group without such disorders (HR 2.10, 95% CI 1.60-2.75 and HR 1.82, 95% CI 1.56-2.12, respectively).

Conclusions: This study suggests potential links between anxiety disorders, age, and mortality among women with 50 years of follow-up, but does not provide definitive conclusions due to the borderline significance of the results.

Strengths and limitations of this study

For peer review only - http://bmjopen.bmj.com/site/about/guidelines.xhtml

- Strengths include a follow-up time of five decades, longitudinal design allows for a comprehensive examination of trends and associations over an extended period.
- The inclusion of a representative sample of middle-aged women in Gothenburg with high participation rates enhances the generalizability of the findings.
- The study considers various confounding factors such as smoking, alcohol consumption, physical activity, socioeconomic status, and educational level, which previous investigations often neglected.
- A psychiatric investigation based on DSM criteria rather than self-assessed symptoms at baseline ensures the accuracy of the disorders.
- Limitations are the relatively small sample size and an exclusively female population.

INTRODUCTION

Worldwide, mental disorders are among the top ten leading causes of morbidity. Major depressive disorder (MDD) and anxiety disorders (AD) are the most significant contributors to this, affecting more than half a billion people, and are overrepresented among women (1,2). The Swedish National Board of Health and Welfare states that persons with mental disorders have an increased risk of incidence as well as mortality in somatic disorders, compared to people without mental illness (3). However, the association between mental disorders and mortality is still debatable. In a Danish population-based cohort study it was seen that mental disorders were associated with a higher mortality rate, regardless of type of mental disorders, and to premature death, both for men and women (4). Also, comorbidity between mental disorder and general medical condition was associated to shorter life expectancy, compared to have one of the conditions only (5). Denollet et al. followed 5000 healthy Dutch women for ten years (6).They found that anxiety symptoms predicted premature all-cause and cardiovascular mortality (6). A later study distinguished between anxiety symptoms and anxiety disorders and followed persons for 11-19 years (7). No prediction of mortality, whether in women or men, was found (7). A Danish register study with nine years of follow-up showed that anxiety disorders significantly increased mortality risk, both in women and men (8). Cardiovascular disease and suicide are both potential contributors to increased mortality among people with MDD and AD (9-11). A variety of factors associated with poor health, such as hypertension, obesity and excess smoking, have also been linked to common mental disorders (12-16). The challenge remains to determine whether depression/anxiety at

For peer review only - http://bmjopen.bmj.com/site/about/guidelines.xhtml

baseline is associated with later smoking, or if smoking is associated with later depression/anxiety (17).

Two meta-analyses from 2015 and 2016 that studied the association between mortality and AD found conflicting results (18,19). One of the meta-analyses concluded that there was an association between mortality and AD, while the other found no such association. The diversity in results could be due to several reasons. Including psychiatric inpatients tended to increase the association (18), while studying a community sample and adjusting for comorbid AD/MDD had the opposite effect (19). Further, the identification of the disorders differed, with the majority based on medical records in one study (18) and self-reported symptoms in the other (19). However, the follow-up time was a median of ten years in one of the meta-analyses but less in the other, which limits the evaluation of the long-term effect. MDD, on the other hand, has repeatedly been associated with increased all-cause mortality, especially among men (20-22). In the Stirling County Study, Murphy et al. showed an association between depression and premature death among men (HR 2.6, 95% CI 1.4-4.9) in 10 years of follow-up. However, this was not seen among depressed women (HR 1.4, 95% CI 0.6-3.2) (23).

In 1968, the Prospective Population Study of Women in Gothenburg started, including middle-aged women (24). Because of its rather extensive content, the repeated follow-up exams and information regarding mortality during more than 50 years, it was possible to study the associations between disorders and mortality from middle age to the elderly. Given the contradictory results in previous research regarding AD and mortality, the aim was primarily to investigate a possible association between all-cause mortality and AD, secondarily MDD and comorbid AD/MDD, respectively. Another aim was to analyse the risk and health factors which influence such a possible association.

METHODS AND MATERIAL

The Population Study of Women in Gothenburg includes a representative sample of 1462 women aged 38, 46, 50, 54 and 60 years old who were invited to a medical examination in 1968-69. The participation rate was 90.1% (24). 899 participants were systematically sampled from the four youngest age groups according to birth date and invited for a psychiatric examination. Nighty-nine participants declined the invitation, and 22 participants were

For peer review only - http://bmjopen.bmj.com/site/about/guidelines.xhtml

excluded because of incomplete data due to language difficulties, psychosis or intellectual disability, leading to a total of 778 participants who fulfilled the psychiatric exam and were included in this study (see Flowchart in Appendix Figure 1). In this subsample, the participation rate was 89% of the invited subjects in the general population. Of those, 22 women were excluded from the present study because of incomplete data due to language difficulties, psychosis or intellectual disability. A comparison between non-participants and participants showed more single women among the non-participants. Still, no differences were found concerning mental disorders or the need for hospitalisation because of them (25,26). The psychiatric investigation was 1-2 hours long and was conducted using a comprehensive structured questionnaire.

Anxiety disorders and Major Depressive Disorder at baseline

A psychiatrist performed the psychiatric examination through a face-to-face interview (27). The findings from the psychiatric investigation were later revised by scrutinising items in the interview questionnaire relevant to a DSM-III MDD diagnosis (25). Cases of AD was identified from the dataset using algorithms based on the DSM-IV criteria. AD included social phobia, panic disorder, agoraphobia, generalised anxiety disorder (GAD) and obsessive-compulsive disorder (OCD). Identifying cases of posttraumatic stress disorder from items in the dataset was impossible. Specific phobias were excluded due to their generally limited clinical importance.

All-cause mortality rate

Information regarding participants' mortality was collected from the Swedish Cause of Death Register. The mortality rate was observed from the first examination date until the 31st of December 2019.

Statistics

Baseline characteristics were described using chi-square and independent t-test. The association between the baseline characteristics in the women with and without AD/MDD was measured by the Relative Risk (RR) and 95% confidence interval.

Poisson regression models were used to analyse associations with mortality, and the effect of assumed risk factors on mortality risk is reported as Hazard Ratio and 95% Confidence

For peer review only - http://bmjopen.bmj.com/site/about/guidelines.xhtml

Interval (HR, 95% CI). This model used is more accurately designated “Poison piecewise exponential model” but will be abbreviated as “Poisson model” in the text.

The follow-up time of each individual is split into many short intervals of equal length. For each interval an event outcome variable is defined, coded 0 for not dead and 1 if not dead in that time interval. This structures survival data as a time-to-event dataset that will for all practical purposes give identical results whether analysed in a Cox model or a Poisson model. An advantage with Poisson models is that age and calendar time can be modelled as continuous risk factors, making it easy to test for interaction with regard to age or calendar time, that is, to test if the effect of a certain (assumed) risk factor varies by age or over calendar years. The hazard ratio (HR, CI 95%) measures the relative risk of death at any arbitrarily chosen time interval that, in the absence of interaction is assumed to be a constant. The risk time was measured from baseline to the time of death or 31st December 2019. The median follow-up time was 36.9 years (minimum 0.33, maximum 51.3 years). The total risk time was 27260 years and subdivided into quarters of a year (91.3 days).
When analysing the association between mortality and all AD and all MDD, covariates were sequentially added (Table 1). Still, each covariate was tested in a separate model with age as the only covariate, as small sample sizes limited the number of covariates that it would be meaningful to enter simultaneously (Table 2).
Statistical significance was accepted at $p < 0.05$ in all analyses except tests of interaction, which was accepted at $p < 0.10$. SPSS version 29 and Stata version 17 were used to perform statistical analysis.

Potential confounders

To reduce the effect of potential confounders, the following covariates, with known influence on mortality, were used: age (in years), education (over or under six years), economic status (defined as annual income divided by number of family members), alcohol consumption (from never to everyday use of wine, beer and liquor, separately, and “regularly” is defined as several days per week or more), smoking (current or non-current), physical activity (“sedentary leisure time” is defined as <4 hours/week)(28) and BMI (weight in kilogram divided with the square of height in meters). Lower educational attainment and income levels have been associated with higher mortality rates (29) and marital status can influence mortality, with married individuals experiencing lower mortality rates compared to those who are unmarried (30).

For peer review only - http://bmjopen.bmj.com/site/about/guidelines.xhtml

All covariates except age were dichotomised in the regression models. These data were collected in interviews or measured at baseline.

RESULTS

AD, MDD and comorbid AD/MDD

One hundred sixty participants (20.6%) had one or more AD, and 57 (7.3%) had MDD in the psychiatric exam 1968-69. Of these, 25 participants (3.2%) had comorbid AD/MDD. The numbers of those with AD solely and MDD solely are presented in Table 1.

The distribution of disorders among AD was GAD n=71 (9.1%), agoraphobia n=67 (8.6%), panic disorder n=58 (7.4%), social phobia n=51 (6.6%) and obsessive-compulsive disorder (OCD) n=28 (3.6%). Of the 135 participants with AD, 85 had one disorder, 48 had two, 17 had three, 7 had four, and 3 had all five disorders.

Baseline characteristics

Baseline characteristics are shown in Table 1. Participants with AD solely (i.e. excluding participants with comorbid AD/MDD) did, to a greater extent, smoke and consume more alcohol (beer) than those without any disorder. Participants with comorbid AD/MDD had significantly more sedentary leisure time and lower economic status. Participants with MDD were, to a lesser extent, married.

Table 1. Baseline characteristics of the different groups of women with AD solely, MDD solely, comorbid AD/MDD and no AD or MDD, at the examination year 1968-69.

**Characteristics, Mean (SD)**	AD solely n=135	MDD solely n=32	Comorbid AD/MDD n=25	No AD or MDD n=586
Age (years)	47.6 (4.4)	47.8 (5.1)	47.2 (5.3)	47.9 (4.5)
Economic status[1], 1000 SEK	16.2 (8.5)	16.6 (12.2)	**12.0 (4.4) ***	16.1 (9.8)
Number of cigarettes per day among smokers	10.5 (6.0)	**14.1 (7.9) ***	11.2 (8.3)	10.2 (6.0)
BMI kg/m2	24.4 (4.3)	23.7 (2.6)	24.2 (3.4)	24.1 (3.7)
**Characteristics, Prevalence (%)**				
Education six years or more, n (%)	32 (23.7)	6 (18.8)	3 (12.0)	153 (26.1)
Marital status, married	102 (75.6)	**19 (59.4) ***	22 (88)	483 (82.4)
Physical activity	27 (20.6)	4 (12.5)	**8 (32.0) ***	95 (16.2)

For peer review only - http://bmjopen.bmj.com/site/about/guidelines.xhtml

Sedentary leisure time[2], n (%)				
Smoking, n (%)	**71 (52.6)** **	16 (50.0)	13 (52.0)	226 (38.8)
Economic status (in 10% lowest income category) n (%)	13 (9.6)	2 (6.3)	1 (4.2)	55 (9.5)
Regular alcohol use, n (%)<br>Beer<br>Wine<br>Spirits	**49 (36.2)** **<br>8 (5.9)<br>1 (0.7)	 5 (15.6)<br>0 (0)<br>0 (0)	 5 (20)<br>0 (0)<br>0 (0)	 162 (27.6)<br>33 (5.6)<br>8 (1.4)
BMI >30	13 (9.6)	1 (3.1)	1 (4.0)	44 (7.5.)
BMI <19	8 (5.9)	1 (3.1)	2 (8.0)	28 (4.8)

* p <0.05 ** p <0.01
[1]Annual income divided by number of family members
[2]<4 hours/week. Values in the cell 'Physical activity Sedentary leisure time' and 'AD solely n=135' mean that 27 women belong to the sedentary category and they comprise 20.6% of those in the AD solely group (5 are missing data on physical activity). The values show the number of persons with a sedentary lifestyle and their proportion in each AD/MDD category.
The significance is tested for the chosen group compared to the group with neither AD nor MDD.

For peer review only

Smoking, beer consumption and physical activity (measured at baseline)

Having AD without MMD significantly increased the relative risk of being a smoker (RR 1.38, CI 1.13-1.67, p<0.01) and regularly consuming beer (RR 1.35, CI 1.05-1.75, p<0.05), compared to not having any of the disorders. Having MDD without AD increased the relative risk of being a heavy smoker (>13 cigarettes/day) (RR 2.23, CI 1.20-4.33, p<0.05). Having comorbid AD/MDD significantly increased the relative risk of having a sedentary leisure time (RR 1.97, CI 1.08-3.59, p=0.05).

All cases of AD and MDD and all-cause mortality rates

Of the total 778 participants, 720 died before the 1st of January 2020. A multivariate regression survival model was used to examine the associations between all cases of AD, all cases of MDD and mortality (Table 2). In the full model, there was no association between AD and mortality. In a corresponding analysis of all cases of MDD, there was a significant association when adjusted for age.

Table 2. Associations between all cases of AD, all cases of MDD and mortality rate in different adjustment models.

	**AD n=160**	**MDD n=57**

Covariates	HR (95% CI)	
Age	**1.25 (1.04-1.49) ***	**1.21 (1.11-1.12) ***
Age, smoking, physical activity, and alcohol use (beer)	**1.20 (1.00-1.44) ***	1.18 (0.90-1.56)
All of the above, economic status, married, education, BMI>30 and BMI<19	1.17 (0.98-1.41)	1.19 (0.90-1.57)
All of the above and depression	1.16 (0.96-1.39)	-
All of the above and anxiety	-	1.15 (0.86-1.52)
Bold figures, statistically significant		

When restricting the analysis (full model including all AD and all MDD) to deaths occurring before the age of 80, AD became a significant risk factor (HR 1.49, CI 1.13-1.98, p<0.01). This association was even stronger in the age range of 65-80 years (HR 1.70, CI 1.26-2.29, p<0.001).

To test the significance of interaction for the effect of AD on mortality in different age intervals, one model with three separate AD-effect parameters was calculated for the age groups 38-64, 65-79 and 80-101. This model was compared to a base model assuming constant AD-effect over the ages. The difference in overall model Chi-square between the two Poisson regression models was 8.2 with df=2 and p=0.02.

An age-adjusted model with only two AD-effect parameters, the first for age 65-79 and the second for the younger and older age intervals pooled, yielded Chi-square= 9.3, df=1 and p=0.002 compared to the base model. A fully adjusted model yielded Chi-square=7.94, df=1 and p=0.005.

When analyzing the relationship between all-cause mortality and MDD, no significant associations were found. However, it's worth noting that the Incidence Rate Ratio (IRR) exhibited a higher magnitude compared to AD, albeit with somewhat less precision.

AD solely, MDD solely, comorbid AD/MDD and all-cause mortality rates

When assessing single risk factors (with age included), a significant association with mortality was found among women with solely AD who smoked. A similar trend was noted in cases of comorbid AD/MDD, as seen in Table 3.

For peer review only - http://bmjopen.bmj.com/site/about/guidelines.xhtml

Table 3. Associations between single covariates and the mortality rate in the total sample and the groups with AD solely, MDD solely, and comorbid AD/ADD. All models include age.

	Total sample n=778	AD solely n=135	MDD solely n=32	Comorbid AD/MDD n=25
Covariate	HR (95% CI)	HR (95% CI)	HR (95% CI)	HR (95% CI)
1. Age	**1.13 (1.12-1.14)** ***	**1.13 (1.11-1.15)** ***	**1.14 (1.09-1.19)** ***	**1.17 (1.11-1.23)** ***
2. Education ≥ 6 years	0.88 (0.74-1.05)	1.00 (0.62-1.60)	1.20 (0.46-3.19)	0.30 (0.04-2.13)
3. Physical activity: Regular exercise	**0.73 (0.63-0.85)** ***	0.71 (0.49-1.04)	0.79 (0.38-1.66)	1.02 (0.40-2.63)
4. Smoking	**1.82 (1.56-2.13)** ***	**1.86 (1.24-2.79)** **	2.12 (0.95-4.72)	**5.18 (1.57-17.14)** **
5. Beer consumption	0.93 (0.84-1.03)	0.83 (0.66-1.05)	0.70 (0.38-1.29)	1.85 (0.76-4.49)
6. BMI<19	**1.66 (1.20-2.31)** ***	2.13 (0.98-4.62)	1.57 (0.17-14.07)	2.38 (0.19-29.8)
7. BMI>30	1.31 (1.00-1.72)	0.88 (0.47-1.67)	2.07 (0.25-16.88)	0.60 (0.07-4.86)
8. Married	1.00 (0.84-1.22)	0.97 (0.62-1.50)	1.29 (0.54-3.07)	1.08 (0.30-3.97)
9. Economic status (lowest decentile)	**1.37 (1.07-1.77)** *	1.43(0.82-2.49)	0.69 (0.22-2.23)	0.37 (0.05-2.86)

In a regression analysis, when adjusted for age, the increased mortality risk associated with smoking did increase further among those having AD, compared to not having AD, HR 2.28 and 1.71, but with overlapping 95% CIs, 1.64-3.17 and 1.44-2.02, respectively, p= 0.096. The corresponding difference in mortality risk associated with regular physical activity was negligible, with HR 0.77 (95% CI = 0.57-1.03) and HR 0.73 (95% CI = 0.62-0.86), p= 0.95.

For peer review only - http://bmjopen.bmj.com/site/about/guidelines.xhtml

DISCUSSION

Main findings

In this 50-year study of middle-aged women, several significant findings emerged. Women with solely AD were more likely to be smokers and beer consumers compared to those without AD or MDD. Notably, women with AD (all cases) exhibited a significantly increased mortality rate when considering age, smoking, physical activity, and beer consumption as covariates in the model. However, this association did not reach statistical significance in the full model. Conversely, women with MDD (all cases) displayed a non-significant trend toward an increased mortality rate in both models. When analyzing deaths specifically within the age range of 65-80 years, a strong and significant association was found between AD and increased mortality rate in. Intriguingly, no such associations were identified for the age intervals of 38-64 and 80-101, indicating a significant interaction effect for AD-Age-Mortality. Notably, no analogous pattern was observed concerning the interaction between MDD-Age-Mortality.

Comparison with other studies

The lack of significant overall association between MDD and all-cause mortality is in contrast to previous findings (20-22), with some exceptions. Holwerda et al. did find an association, but only among elderly men (22). Eaton et al., on the other hand, did not find any association, and the lack of this is suggested to be primarily due to a long follow-up time (27 years). Symptoms of MDD could be disguised signs of poor health. In the short term, this could lead to an increase in mortality risk that later would subside (31). However, if this were applicable in our study, it would have been revealed in our sample when analysing time intervals, which was not the case.

Our sample of women with MDD may have been too small, which may explain the lack of significant findings in this group. It is notable, however, that in most of our analyses of MDD and mortality, the hazard ratios were similar to those in the analyses of AD. For example, in the 80-101 age range the hazard ratio for MMD was 1.39 (95% CI 0.98-1.98).

The finding of a reduced association between AD and mortality rate when the full model was applied is consistent with previous research (19,21,31). The increased mortality rate among women with AD in the age range 65-80 is not readily comparable to earlier studies due to the difference in follow-up time. One interpretation of this finding could be that AD is associated

For peer review only - http://bmjopen.bmj.com/site/about/guidelines.xhtml

with mortality only in ages >65 years. This contradicts previous results, where AD in older women has not been associated with increased mortality but concurs with our finding of no association after age 80 (22,32). Another interpretation is that AD that lasts for an extended period of time, increases vulnerability and may impede the ability of individuals to manage their health and access necessary medical care, which can further contribute to an increased morbidity and mortality risk.

Smoking is an established risk factor for mortality. The association between smoking and mortality tended to increase further in the presence of AD and comorbid AD/MDD. A different pattern in smoking habits could explain this. We found that smoking was more common among women with AD. Another study reported that persons with AD, to a lesser extent, cease daily smoking. If they do, the duration of smoking is longer (33). Our study did not investigate the exposure time. Still, if our sample is comparable to that study, and the women had a longer duration of smoking, this could be one possible contributing cause to the late increase in mortality.

A meta-analysis of smoking and mortality found that the mortality rate decreased among female smokers after 75 or 80 years (34). This is consistent with our findings of reduced mortality after 80 years among women with AD, where 53% were smoking at baseline. One possible explanation is that after the age of 80 years, subjects susceptible to harmful smoking effects may have died already. Another possible reason might be that after the age of 80 years, other causes of death increase and compete with those related to smoking and AD.

Strengths and weaknesses

This study exhibits several strengths, including its extended five-decade longitudinal design, a representative sample of middle-aged women in Gothenburg with high participation rates, and a comprehensive consideration of various confounding factors. Importantly, prior investigations reporting increased mortality rates in anxiety disorders did not control for covariates like smoking, alcohol consumption, physical activity, socioeconomic status, or educational level, which could have influenced their findings (4,5,8).

Also, many studies investigating AD and MDD have used various self-administrated questionnaires, measuring symptomatology rather than the frequency of a disorder. This could

For peer review only - http://bmjopen.bmj.com/site/about/guidelines.xhtml

potentially over- or underdiagnose the psychopathology, and the reported symptoms could originate from a somatic condition. In this study, however, the disorder was assessed by a psychiatrist, making the results more accurate. Furthermore, studies tend to investigate associations either among persons with AD or MDD, which could be misleading due to the high comorbidity. In contrast, in the current study the participants with comorbid AD/MDD were examined as a separate group.

The study has some limitations. Firstly, the research sample was relatively small, especially regarding the group having MDD and comorbid AD/MDD. While there is a potential for complex causal mechanisms linking depression status, lifestyle factors such as smoking and alcohol consumption, and mortality risks, our study is primarily focused on examining associations and variations. Introducing complex models would not enhance our analysis, as our dataset lacks the necessary scope to support such an approach. Another limitation was that the study sample only included women. Furthermore, somatic diseases among the participants were not taken into account. It is possible that patients with AD could have a higher rate of somatic disease, which in turn could affect the mortality rate. Lastly, it's important to acknowledge that the decision made at the outset to confine the evaluation of baseline risk factors and their outcomes, as well as to assess the point prevalence of AD and MDD (not the lifetime prevalence), represents a limitation in this study. However, it's worth noting that this limitation may not necessarily introduce biases, especially when taking into account the specific research focus of our study. However, the onset of AD is, above all, in early adolescence to young adulthood and often has a long duration, which is why we can consider that the participants who meet the criterion for AD in middle age have had the disorder for a long time. Correspondingly, most of those with the preponderance to incur the condition should have received it by the time of the baseline examination. The same is true for MDD, which has a median onset in young adulthood (35).

The significance of the study, unanswered questions and future research

This study adds to the previous findings showing that AD is not convincingly associated with mortality over the entire age interval studied. The same applies to comorbid AD/MDD. The MDD group was small, however, and the results concerning MDD and mortality are therefore inconclusive. The increase in mortality among those with AD aged 65-80 years is a novel finding that needs to be confirmed in future studies. Considering the high prevalence of comorbid AD/MDD cases, future studies on the association between mortality and AD or

For peer review only - http://bmjopen.bmj.com/site/about/guidelines.xhtml

MDD should include both disorders. Future research should also focus on effective methods to promote a healthier lifestyle among persons with mental disorders especially regarding cessation of smoking and reduction of heavy alcohol consumption to counteract the increased likelihood of developing comorbid medical conditions such as cardiovascular disease.

Funding

This work was supported by Grants from the Swedish Research Council for Health, Working Life and Welfare—FORTE 2007-1958 and grants from the Swedish State under the agreement between the Swedish Government and the county council, the ALF agreement GBG-68771. This research received no specific grant from any public, commercial or not-for-profit sector funding agency.

Competing interests

None declared.

Each author's contribution

The study was conducted as part of AvBs intern research project. DH, TH and CB supervised the work. TH was responsible for the AD and MDD diagnostic procedures. AvB analysed the data and prepared the draft manuscript with the support of all the other authors, DH, TH and CB. VS devised the statistical analysis and contributed to the interpretation of it. All the authors approved the final manuscript and revision.

Data sharing statement

Data are available on reasonable request. All data relevant to the study are included in the article.

Ethics Approval Statement

The Regional Ethical Committee in Gothenburg approved the study (258-16 T853-16). The research was conducted following the World Medical Association Declaration of Helsinki, and all participants provided written informed consent. The manuscript does not contain personal and/or medical information about an identifiable individual. The manuscript is sufficiently anonymised in line with BMJ Open anonymisation policy and checklist. All data are analysed on group level.

For peer review only - http://bmjopen.bmj.com/site/about/guidelines.xhtml

For peer review only

REFERENCES

1. GBD 2019 Mental Disorders Collaborators. Global, regional, and national burden of 12 mental disorders in 204 countries and territories, 1990-2019: a systematic analysis for the Global Burden of Disease Study 2019. *Lancet Psychiatry*. 2022;9:137-150. doi:10.1016/S2215-0366(21)00395-3

2. Dattani S, Ritchie H. Roser M. "Mental Health". Published online at OurWorldInData.org. 2021.Retrieved from: 'https://ourworldindata.org/mental-health' [Online Resource] May 9, 2023

3. National Board of Health and Welfare, Sweden. Evaluation of care at depression and anxiety disorders. National guidelines – Evaluation Main report with areas for improvement (Socialstyrelsen. Utvärdering av vård vid depression och ångestsyndrom Nationella riktlinjer – Utvärdering. Huvudrapport med förbättringsområden) https://www.socialstyrelsen.se/globalassets/sharepointdokument/artikelkatalog/nationella-riktlinjer/2019-5-12.pdf. 2019.

4. Plana-Ripoll O, Pedersen CB, Agerbo E, Holtz Y, Erlangsen A, Canudas-Romo V, et al. A comprehensive analysis of mortality-related health metrics associated with mental disorders: a nationwide, register-based cohort study. Lancet. 2019;394(10211):1827-35.

5. Momen NC, Plana-Ripoll O, Agerbo E, Christensen MK, Iburg KM, Laursen TM, et al. Mortality Associated With Mental Disorders and Comorbid General Medical Conditions. JAMA Psychiatry. 2022;79(5):444-53.

6. Denollet J, Maas K, Knottnerus A, Keyzer JJ, Pop VJ. Anxiety predicted premature all-cause and cardiovascular death in a 10-year follow-up of middle-aged women. *Journal of clinical epidemiology*. 2009;62:452-456. doi:10.1016/j.jclinepi.2008.08.006

7. Saeed Mirza S, Ikram MA, Hofman A, Tiemeier H. Anxiety does not predict mortality. A population-based study. *World Psychiatry*. 2015;14:103-104. doi:10.1002/wps.20192

8. Meier SM, Mattheisen M, Mors O, Mortensen PB, Laursen TM, Penninx BW. Increased mortality among people with anxiety disorders: total population study. *Br J Psychiatry*. 2016;209:216-221. doi:10.1192/bjp.bp.115.171975

9. Roest AM, Martens EJ, de Jonge P, Denollet J. Anxiety and risk of incident coronary heart disease: a meta-analysis. *J Am Coll Cardiol*. 2010;56:38-46. doi:10.1016/j.jacc.2010.03.034

10. Van der Kooy K, van Hout H, Marwijk H, Marten H, Stehouwer C, Beekman A. Depression and the risk for cardiovascular diseases: systematic review and meta analysis. *Int J Geriatr Psychiatry*. 2007;22:613-626. doi:10.1002/gps.1723

11. Moitra M, Santomauro D, Degenhardt L, et al. Estimating the risk of suicide associated with mental disorders: A systematic review and meta-regression analysis. *J Psychiatr Res*. 2021;137:242-249. doi:10.1016/j.jpsychires.2021.02.053

12. Luppino FS, de Wit LM, Bouvy PF, et al. Overweight, obesity, and depression: a systematic review and meta-analysis of longitudinal studies. *Arch Gen Psychiat*. 2010;67:220-9. doi:10.1001/archgenpsychiatry.2010.2

13. Konttinen H, van Strien T, Mannisto S, Jousilahti P, Haukkala A. Depression, emotional eating and long-term weight changes: a population-based prospective study. *Int J Behav Nutr Phys Act*. 2019;16:28. doi:10.1186/s12966-019-0791-8

For peer review only - http://bmjopen.bmj.com/site/about/guidelines.xhtml

14. Brumpton B, Langhammer A, Romundstad P, Chen Y, Mai XM. The associations of anxiety and depression symptoms with weight change and incident obesity: The HUNT Study. *Int J Obes (Lond)*. 2013;37:1268-74. doi:10.1038/ijo.2012.204
15. Lim LF, Solmi M, Cortese S. Association between anxiety and hypertension in adults: A systematic review and meta-analysis. *Neurosci Biobehav Rev*. 2021;131:96-119. doi:10.1016/j.neubiorev.2021.08.031
16. Weinberger AH, Chaiton MO, Zhu J, Wall MM, Hasin DS, Goodwin RD. Trends in the Prevalence of Current, Daily, and Nondaily Cigarette Smoking and Quit Ratios by Depression Status in the U.S.: 2005-2017. *Am J Prev Med*. 2020;58:691-698. doi:10.1016/j.amepre.2019.12.023
17. Fluharty M, Taylor AE, Grabski M, Munafo MR. The Association of Cigarette Smoking With Depression and Anxiety: A Systematic Review. *Nicotine Tob Res*. 2017;19:3-13. doi:10.1093/ntr/ntw140
18. Walker ER, McGee RE, Druss BG. Mortality in mental disorders and global disease burden implications: a systematic review and meta-analysis. *JAMA Psychiatry*. 2015;72:334-41. doi:10.1001/jamapsychiatry.2014.2502
19. Miloyan B, Bulley A, Bandeen-Roche K, Eaton WW, Goncalves-Bradley DC. Anxiety disorders and all-cause mortality: systematic review and meta-analysis. *Soc Psychiatry Psychiatr Epidemiol*. 2016;51:1467-1475. doi:10.1007/s00127-016-1284-6
20. van Dijk MR, Utens EM, Dulfer K, et al. Depression and anxiety symptoms as predictors of mortality in PCI patients at 10 years of follow-up. *Eur J Prev Cardiol*. 2016;23:552-558. doi:10.1177/2047487315571889
21. Mykletun A, Bjerkeset O, Overland S, Prince M, Dewey M, Stewart R. Levels of anxiety and depression as predictors of mortality: the HUNT study. *Br J Psychiatry*. 2009;195:118-125. doi:10.1192/bjp.bp.108.054866
22. Holwerda TJ, Schoevers RA, Dekker J, Deeg DJ, Jonker C, Beekman AT. The relationship between generalized anxiety disorder, depression and mortality in old age. *Int J Geriatr Psychiatry*. 2007;22:241-249. doi:10.1002/gps.1669
23. Murphy JM, Gilman SE, Lesage A, et al. Time trends in mortality associated with depression: findings from the Stirling County study. *Can J Psychiatry*. 2010;55:776-783. doi:10.1177/070674371005501205
24. Bengtsson C, Blohme G, Hallberg L, et al. The study of women in Gothenburg 1968-1969-a population study. General design, purpose and sampling results. *Acta medica Scandinavica*. 1973;193:311-318.
25. Hällström T. Point prevalence of major depressive disorder in a Swedish urban female population. *Acta Psychiatr Scand*. 1984;69:52-9. doi:10.1111/j.1600-0447.1984.tb04516.x
26. Hällström T. *Mental disorder and sexuality in the climacteric : a study in psychiatric epidemiology*. Scandinavian University Books, Esselte Studium, Gothenburg. Diss. Gothenburg : Univ.; 1973.
27. Åsberg M, Montgomery SA, Perris C, Schalling D, Sedvall G. A comprehensive psychopathological rating scale. *Acta Psychiatr Scand Suppl*. 1978:5-27. doi:10.1111/j.1600-0447.1978.tb02357.x
28. Saltin B, Grimby G. Physiological analysis of middle-aged and old former athletes. Comparison with still active athletes of the same ages. *Circulation*. 1968;38:1104-1115. doi:10.1161/01.cir.38.6.1104

For peer review only - http://bmjopen.bmj.com/site/about/guidelines.xhtml

29. Backlund E, Sorlie PD, Johnson NJ. The shape of the relationship between income and mortality in the United States. Evidence from the National Longitudinal Mortality Study. Annals of epidemiology. 1996;6(1):12-20; discussion 1-2.
30. Manzoli L, Villari P, G MP, Boccia A. Marital status and mortality in the elderly: a systematic review and meta-analysis. Social science & medicine (1982). 2007;64(1):77-94.

31. Eaton WW, Roth KB, Bruce M, et al. The relationship of mental and behavioral disorders to all-cause mortality in a 27-year follow-up of 4 epidemiologic catchment area samples. *American journal of epidemiology*. 2013;178:1366-77. doi:10.1093/aje/kwt219
32. van Hout HP, Beekman AT, de Beurs E, et al. Anxiety and the risk of death in older men and women. *Br J Psychiatry*. 2004;185:399-404. doi:10.1192/bjp.185.5.399
33. Lawrence D, Considine J, Mitrou F, Zubrick SR. Anxiety disorders and cigarette smoking: Results from the Australian Survey of Mental Health and Wellbeing. *Aust N Z J Psychiatry*. 2010;44:520-527. doi:10.3109/00048670903571580
34. Gellert C, Schottker B, Brenner H. Smoking and all-cause mortality in older people: systematic review and meta-analysis. *Arch Intern Med*. 2012;172:837-844. doi:10.1001/archinternmed.2012.1397
35. Solmi M, Radua J, Olivola M, et al. Age at onset of mental disorders worldwide: large-scale meta-analysis of 192 epidemiological studies. *Mol Psychiatry*. 2022;27:281-295. doi:10.1038/s41380-021-01161-7

For peer review only

For peer review only - http://bmjopen.bmj.com/site/about/guidelines.xhtml

Appendix Figure 1

Flowchart of participants in Population Study of Women in Gothenburg in 1968-69

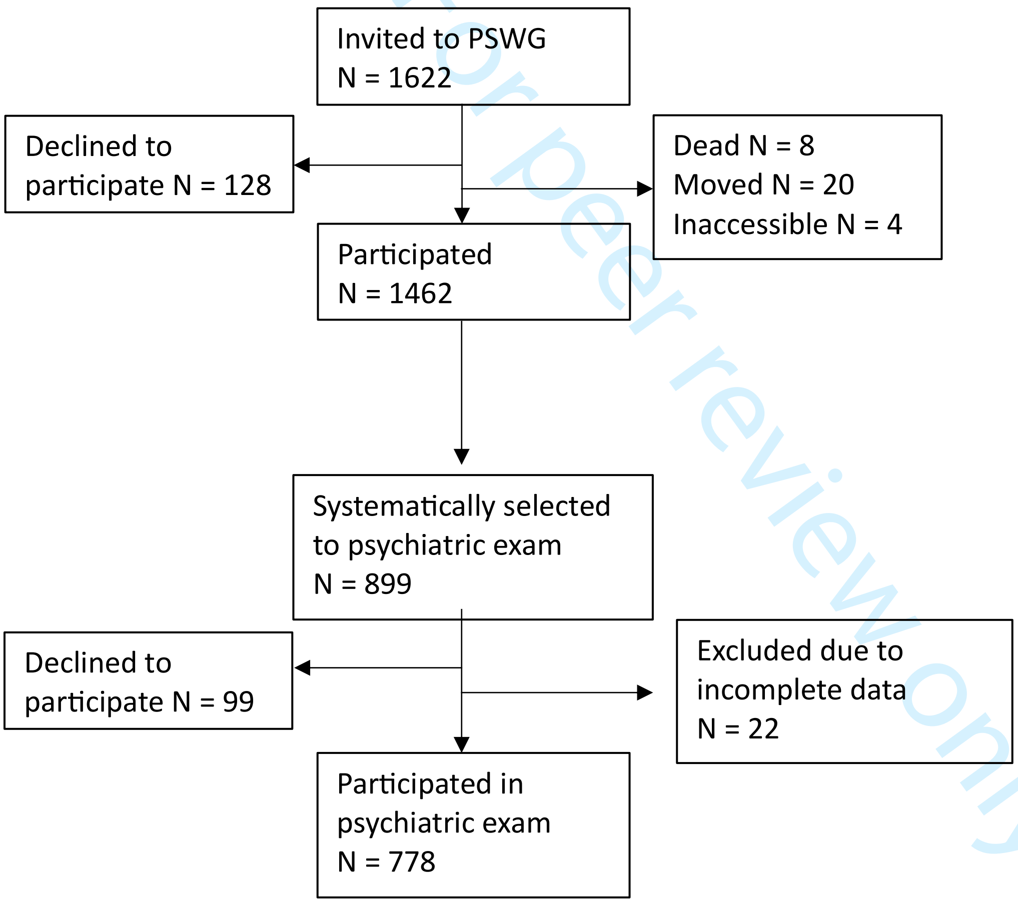

For peer review only

Reporting checklist for cohort study.

Based on the STROBE cohort guidelines.

Instructions to authors

Complete this checklist by entering the page numbers from your manuscript where readers will find each of the items listed below.

Your article may not currently address all the items on the checklist. Please modify your text to include the missing information. If you are certain that an item does not apply, please write "n/a" and provide a short explanation.

Upload your completed checklist as an extra file when you submit to a journal.

In your methods section, say that you used the STROBE cohortreporting guidelines, and cite them as:

von Elm E, Altman DG, Egger M, Pocock SJ, Gotzsche PC, Vandenbroucke JP. The Strengthening the Reporting of Observational Studies in Epidemiology (STROBE) Statement: guidelines for reporting observational studies.

		Reporting Item	Page Number
**Title and abstract**			
Title	#1a	Indicate the study's design with a commonly used term in the title or the abstract	1
Abstract	#1b	Provide in the abstract an informative and balanced summary of what was done and what was found	2
**Introduction**			
Background / rationale	#2	Explain the scientific background and rationale for the investigation being reported	3
Objectives	#3	State specific objectives, including any prespecified hypotheses	4
**Methods**			
Study design	#4	Present key elements of study design early in the paper	4

For peer review only

Setting	#5	Describe the setting, locations, and relevant dates, including periods of recruitment, exposure, follow-up, and data collection	4
Eligibility criteria	#6a	Give the eligibility criteria, and the sources and methods of selection of participants. Describe methods of follow-up.	4
Eligibility criteria	#6b	For matched studies, give matching criteria and number of exposed and unexposed	n/a
Variables	#7	Clearly define all outcomes, exposures, predictors, potential confounders, and effect modifiers. Give diagnostic criteria, if applicable	5
Data sources / measurement	#8	For each variable of interest give sources of data and details of methods of assessment (measurement). Describe comparability of assessment methods if there is more than one group. Give information separately for for exposed and unexposed groups if applicable.	5
Bias	#9	Describe any efforts to address potential sources of bias	n/a
Study size	#10	Explain how the study size was arrived at	4
Quantitative variables	#11	Explain how quantitative variables were handled in the analyses. If applicable, describe which groupings were chosen, and why	5
Statistical methods	#12a	Describe all statistical methods, including those used to control for confounding	
5			
Statistical methods	#12b	Describe any methods used to examine subgroups and interactions	5
Statistical methods	#12c	Explain how missing data were addressed	5
Statistical methods	#12d	If applicable, explain how loss to follow-up was addressed	5
Statistical methods	#12e	Describe any sensitivity analyses	
5			

Results

For peer review only

Participants	#13a	Report numbers of individuals at each stage of study—eg numbers potentially eligible, examined for eligibility, confirmed eligible, included in the study, completing follow-up, and analysed. Give information separately for for exposed and unexposed groups if applicable.	6
Participants	#13b	Give reasons for non-participation at each stage	5
Participants	#13c	Consider use of a flow diagram	
n/a			
Descriptive data	#14a	Give characteristics of study participants (eg demographic, clinical, social) and information on exposures and potential confounders. Give information separately for exposed and unexposed groups if applicable.	6
Descriptive data	#14b	Indicate number of participants with missing data for each variable of interest	
n/a			
Descriptive data	#14c	Summarise follow-up time (eg, average and total amount)	
6			
Outcome data	#15	Report numbers of outcome events or summary measures over time. Give information separately for exposed and unexposed groups if applicable.	
7			
Main results	#16a	Give unadjusted estimates and, if applicable, confounder-adjusted estimates and their precision (eg, 95% confidence interval). Make clear which confounders were adjusted for and why they were included	8
Main results	#16b	Report category boundaries when continuous variables were categorized	n/a
Main results	#16c	If relevant, consider translating estimates of relative risk into absolute risk for a meaningful time period	
8			

For peer review only

Other analyses	#17	Report other analyses done—eg analyses of subgroups and interactions, and sensitivity analyses	n/a
**Discussion**			
Key results	#18	Summarise key results with reference to study objectives	10
Limitations	#19	Discuss limitations of the study, taking into account sources of potential bias or imprecision. Discuss both direction and magnitude of any potential bias.	12
Interpretation	#20	Give a cautious overall interpretation considering objectives, limitations, multiplicity of analyses, results from similar studies, and other relevant evidence.	12
Generalisability	#21	Discuss the generalisability (external validity) of the study results	12
**Other Information**			
Funding	#22	Give the source of funding and the role of the funders for the present study and, if applicable, for the original study on which the present article is based	12

The STROBE checklist is distributed under the terms of the Creative Commons Attribution License CC-BY. This checklist was completed on 09. May 2023 using https://www.goodreports.org/, a tool made by the EQUATOR Network in collaboration with Penelope.ai

For peer review only
